# Micro-osteoperforation for accelerating orthodontic tooth movement: a meta-analysis of short-term efficacy, safety, and optimal application parameters

**DOI:** 10.3389/fdmed.2026.1814419

**Published:** 2026-05-14

**Authors:** Shu-Hong Ching, Xiaozhe Wang, Danyang Li, Qianhan Zheng, Jiahao Chen, Yi Zheng, Xuepeng Chen, Qian Liu

**Affiliations:** Stomatology Hospital, School of Stomatology, Zhejiang University School of Medicine, Clinical Research Center for Oral Diseases of Zhejiang Province, Key Laboratory of Oral Biomedical Research of Zhejiang Province, Cancer Center of Zhejiang University, Hangzhou, China

**Keywords:** acceleratory orthodontic interventions, anchorage changes, micro-osteoperforation, orthodontics, pain and life quality, periodontal parameters, root resorption, tooth movement techniques

## Abstract

**Background:**

Prolonged orthodontic treatment is associated with adverse clinical outcomes such as enamel white spot lesions and root resorption, and micro-osteoperforation (MOPs) is a minimally invasive intervention for accelerating orthodontic tooth movement (OTM).

**Objectives:**

This meta-analysis aimed to evaluate the efficacy of MOPs in accelerating OTM, assess its potential adverse effects on periodontal tissues, root structure and anchorage, and summarize the optimal application parameters of MOPs for clinical practice.

**Methods:**

A systematic literature search was conducted in PubMed, Web of Science, EMBASE, Scopus, Cochrane Library, LILACS, and Google Scholar, with 16 randomized controlled trials (RCTs) included after screening 1,175 records. This study was registered in the PROSPERO International Prospective Register of Systematic Reviews (registration number: CRD420251149503). The Cochrane ROB 2 tool was used to assess the risk of bias of included studies, and the GRADE approach was applied to evaluate the certainty of the cumulative evidence.

**Results:**

Compared with traditional orthodontic treatment, the MOPs group achieved an increase of 0.67 mm (95% CI: 0.45–0.88) in the total OTM distance and a monthly increase of 0.20 mm (95% CI: 0.13–0.26) in the OTM rate. Multiple MOPs significantly increased the total OTM distance (1.17 mm, 95% CI: 0.21–2.13) compared with single MOPs (0.61 mm, 95% CI: 0.45–0.77), while the monthly OTM rate remained stable at approximately 0.2 mm. For secondary outcomes, MOPs was associated with a non-clinically significant increase in root resorption (mean difference: 0.19 mm, 95% CI: −0.14 to 0.51), no significant anchorage loss, and mild, transient adverse effects on periodontal parameters (e.g., slight increase in probing depth). Postoperative pain in the MOPs group was significantly higher on the surgery day but diminished rapidly, with no long-term impact on patients’ quality of life. Subgroup analyses showed that mandibular MOPs, repeated MOPs application, and the use of the Propel device were associated with enhanced acceleration effects.

**Risk of bias:**

The included studies had an overall low risk of bias in randomization, incomplete outcome data and selective reporting domains. Moderate risks of performance and detection bias were present across all studies due to the inherent inability to blind participants and operators for surgical interventions such as MOPs.

**Conclusions:**

MOPs appears to be an effective adjunctive approach for accelerating orthodontic tooth movement without causing clinically significant damage to root structure, anchorage, or periodontal tissues. Meanwhile, long-term outcomes and optimal application parameters—such as the number, depth, and frequency of perforations—require further investigation. Clinical implementation should be individualized based on patient characteristics and treatment objectives.

**Systematic Review Registration:**

https://www.crd.york.ac.uk/PROSPERO/recorddashboard, PROSPERO CRD420251149503.

## 1 Introduction

Prolonged orthodontic treatment duration constitutes a major clinical challenge, as it may lead to patient refusal of orthodontic intervention and contribute to the development of enamel white spot lesions, dental caries, and root resorption. In response, increasing attention has been directed toward novel and efficient approaches to accelerate tooth movement. The concept of corticotomy, which aimed to achieve faster tooth movement, was introduced by Kole in 1959 (1). Following the proposal of the regional accelerated phenomenon (RAP) by Frost in 1983 (2), clinicians and researchers gradually accepted that trauma-induced rapid alveolar bone reconstruction could accelerate orthodontic tooth movement. Subsequently, other innovative surgical techniques and concepts—such as corticision, surgically facilitated orthodontic therapy (SFOT), corticotomy-assisted orthodontic treatment (CAOT), and periodontally accelerated osteogenic orthodontics (PAOO)—were introduced in the following years. Although these surgical approaches significantly shorten orthodontic treatment duration (3), they are associated with notable postoperative reactions and discomfort (e.g., flap surgery-induced alveolar bone crest resorption, gingival recession, pain, and swelling). Moreover, due to their high technical sensitivity, these techniques remain underutilized in routine clinical practice. Consequently, several more minimally invasive and digitally assisted surgical methods have been developed, including Piezocision, piezopuncture, laser-assisted flapless corticotomy (LAFC), and micro-osteoperforation (MOPs) (4). Among these, MOPs exhibits superior minimally invasive characteristics (4, 5).

Micro-osteoperforation accelerates orthodontic tooth movement based on the RAP principle. It's typical steps include preoperative evaluation, minimally invasive incision and tissue separation, targeted bone perforations in the alveolar bone, and surgical site cleaning and closure. By creating small perforations in the alveolar bone, MOPs triggers a controlled cytokine and inflammatory cascade, enhances osteoclast activity, and promotes bone remodeling at the target site, thereby accelerating tooth displacement and shortening orthodontic duration (6–8). Research indicates that MOPs can significantly reduce orthodontic treatment time without the need for more invasive surgeries. Some studies have also shown that, compared with traditional invasive techniques such as corticotomy, PAOO, and Piezocision, MOPs achieves a comparable acceleration effect, although multiple MOPs procedures may be required in some cases. Moreover, several studies have reported that MOPs does not compromise orthodontic anchorage or increase the risk of root resorption—a concern of great importance to both patients and clinicians—and such adverse events have not been consistently reported across all MOPs-related clinical trials. Compared with conventional surgeries, MOPs is associated with mild discomfort and fewer postoperative reactions, making it more acceptable to orthodontic patients. In addition, the technique is straightforward, requires a short operation time, and involves minimal instrumentation (e.g., miniscrew anchorage or the Propel device; Propel Orthodontics, Ossining, USA), enabling general orthodontists to perform it in routine clinical practice (8–10). Nevertheless, some studies have concluded that MOPs does not accelerate tooth movement and have reported root resorption of the first premolar.

Furthermore, the location of MOPs varies depending on the target teeth in the maxilla and mandible. Whether MOPs is performed using standard Propel instruments and whether single or multiple perforations are applied may also influence study outcomes, contributing to heterogeneity across recent studies. Meanwhile, not all potential risks associated with MOPs have been fully elucidated. Postoperative periodontal conditions have been reported in only a few studies. Damage to tooth roots during the drilling process has not been documented in the literature, and pulp status remains largely unexamined in most studies (11–13). Although several literature reviews and meta-analyses in recent years have focused on the efficacy of MOPs in accelerating orthodontic tooth movement, these reviews were published relatively early, applied inconsistent inclusion criteria, and did not comprehensively summarize the adverse effects related to periodontal tissues.

Therefore, the present meta-analysis aims to comprehensively collect high-quality randomized controlled trials published within the last six years to thoroughly evaluate the effectiveness of MOPs in accelerating tooth movement, its impact on root resorption, and its potential adverse effects on periodontal tissues and pain experience. This study seeks to provide additional evidence-based support for clinical decision-making and practice in dentistry.

## 2 Methods

This meta-analysis was conducted in accordance with the Preferred Reporting Items for Systematic Reviews and Meta-Analyses (PRISMA) 2020 guidelines and was prospectively registered in the PROSPERO International Prospective Register of Systematic Reviews (registration number: CRD420251149503).

### 2.1 Study selection

This meta-analysis was based on the PICOS model, focusing on orthodontic patients (Population), evaluating the different effect of MOPs (Intervention) and traditional conventional orthodontics (Comparison). The primary outcomes assessed included the distance of tooth movement and the rate of tooth movement, the secondary outcomes included side effects such as root resorption, anchorage changes, periodontal conditions, pain and life quality (Outcome) in this systematic review and meta-analysis (Study design).

In addition to the risk of bias assessment, the Grading of Recommendations, Assessment, Development and Evaluations (GRADE) approach was used to evaluate the certainty of the cumulative evidence for the primary (tooth movement distance and rate) and secondary outcomes (root resorption, anchorage loss, periodontal parameters, pain and quality of life). The evidence certainty was classified into four levels: high, moderate, low, and very low, based on five domains (risk of bias, inconsistency, indirectness, imprecision, and publication bias).

### 2.2 Search strategy

We conducted a literature search in December 2025 with the following electronic databases: PubMed, Web of Science, EMBASE, Scopus, Web of Science, Cochrane Library, LILACS and Google Scholar. The query parameters contain the following key words: orthodontic tooth movement, canine retraction, tooth movement, teeth movement, root resorption, periodontal tissue, pain, osteogenesis, Tooth Movement Techniques, micro-osteoperforation, micro-perforation, flapless osteopuncture and MOPs. The detailed search strategies were shown in Supplementary Materials Method 1.

### 2.3 Inclusion and exclusion criteria

Research articles were considered eligible when they satisfied all the following requirements: 1. The publication was written in English. 2. The participants were receiving fixed orthodontic treatment and displayed teeth with normal root shapes, free from internal or external resorption, pronounced root curvature, or ankylosis. 3. MOPs served as the main intervention technique evaluated. 4. The complete article text was available for review. Studies were excluded if they met one or more of the conditions below: 1. Inclusion of individuals who had previously undergone orthodontic therapy. 2. Enrollment of participants presenting systemic disorders, active smoking habits, pregnancy, periodontal pathology, or inadequate oral hygiene. 3. Reports in the form of single cases or small case series.

### 2.4 Study selection

Two authors (SHC and XW) carried out independent searches using predetermined keywords within the titles and abstracts of articles identified from the selected databases. Any disagreements in identifying eligible studies were settled through discussion with the third author (DL) until a mutual agreement was achieved. The search outputs were uploaded into EndNote, where duplicate entries were systematically removed. Subsequently, SHC and XW independently reviewed the titles and abstracts to eliminate studies irrelevant to the topic. For articles deemed potentially suitable, the full texts were retrieved and examined in detail by the same two reviewers.

### 2.5 Data collection process

Data extraction was independently conducted by two authors (SHC and XW). The extracted information included study characteristics (author, year and study design), participant demographics (sample size, sex distribution and age), clinical parameters (type of malocclusion, retraction model, MOPs protocol, use of the Propel system), and study follow-up details (duration, drop-outs). Key clinical outcomes were recorded, encompassing the rate of tooth movement, periodontal health, pain or discomfort, root resorption, anchorage loss, methods of outcome measurement and final conclusions.

### 2.6 Synthesis of results

Meta-analyses were performed using a random-effects model to account for potential heterogeneity across studies. Heterogeneity was quantified using the I^2^ statistic, with I^2^ > 75% defined as high heterogeneity (14). To explore the primary drivers of heterogeneity, a meta-regression analysis was conducted with the following pre-specified covariates: patient age (mean age of participants), tooth type (canine vs. incisor vs. en-masse retraction), perforation frequency (single vs. multiple), perforation depth (3–5 mm vs. 6–7 mm), and instrument type (Propel vs. other instruments). Statistical significance for meta-regression was set at *P* < 0.05. For subgroup analyses, both 95% confidence intervals (CI) and 95% prediction intervals (PI) were reported; PI was used to estimate the range of expected effects in future clinical settings (15).

### 2.7 Risk of bias and applicability assessment

Bias and applicability were assessed by two independent reviewers with the ROB 2 tool (Cochrane's Risk of Bias Tool). The tool assesses seven domains as follows: (1) bias in random sequence generation; (2) bias in allocation concealment; (3) bias in blinding of participants and personnel; (4) bias in blinding of outcome assessment; (5) bias due to incomplete outcome data; (6) bias in selective reporting; (7) other bias. Studies were ultimately categorized as “low risk of bias”, “high risk of bias” or “unclear”.

## 3 Results

### 3.1 Study selection and characteristic of studies

**Figure 1:**
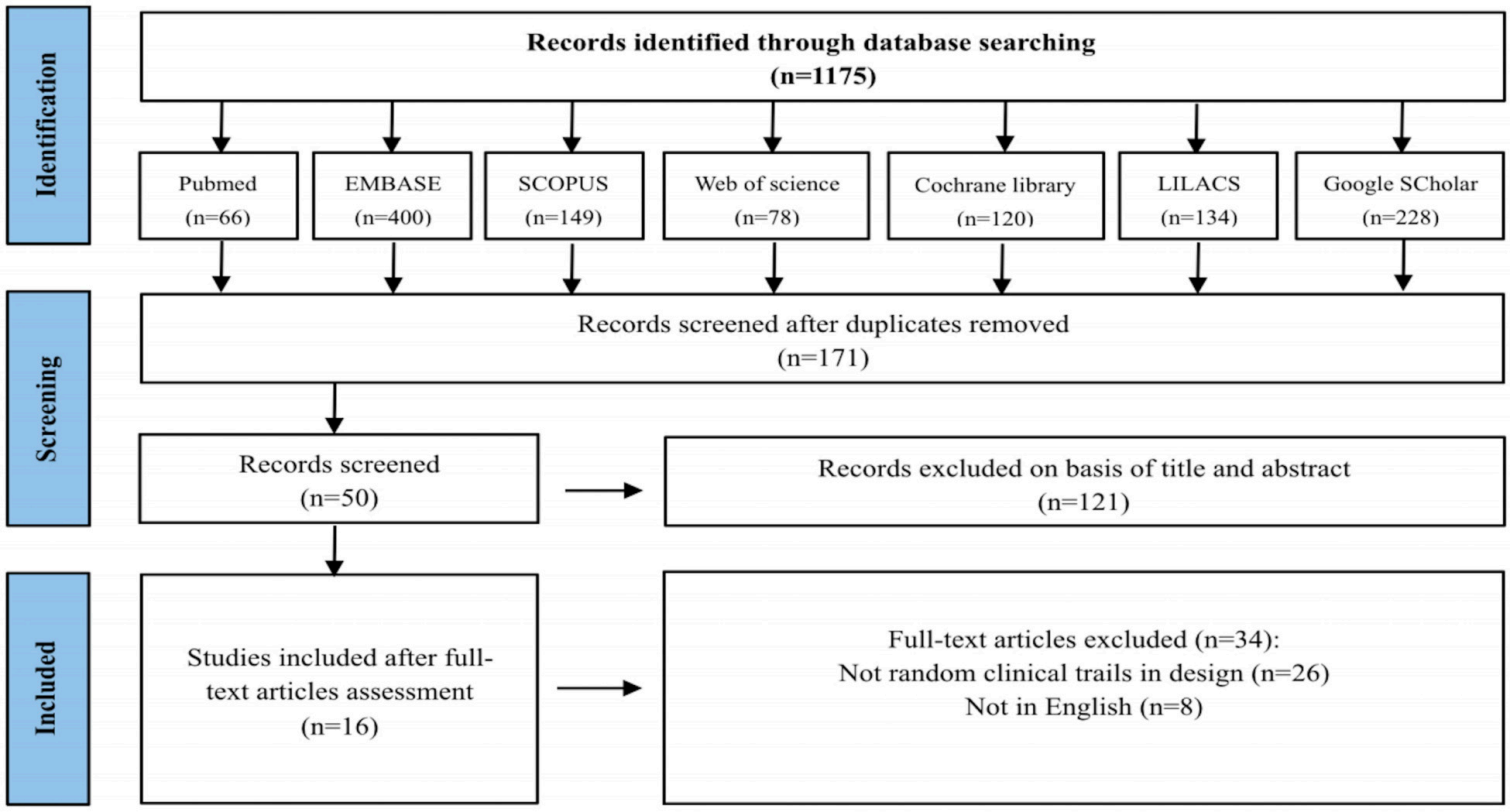
PRISMA 2020n flow diagram of the study selection process. This diagram illustrates the full workflow of our literature screening: 1,175 initial records were identified from 7 electronic databases; after removing duplicates, 171 records were screened; 50 full-text articles were assessed for eligibility; finally, 16 randomized controlled trials were included in this meta-analysis, with detailed reasons for exclusions at each stage.

A total of 1,175 articles were retrieved: 66 from PubMed, 400 from EMBASE, 149 from Scopus, 78 from Web of Science, 120 from the Cochrane Library, 134 from LILACS, and 228 from Google Scholar (Figure 1). After removing 1,004 duplicates, the titles and abstracts of 171 articles were screened. Of these, 50 studies were identified for full-text review. Following detailed evaluation, 26 studies were excluded due to non-randomized controlled trial designs, and 8 were excluded for not being published in English. Ultimately, 16 articles met the inclusion criteria and were included in the qualitative synthesis.

The 16 RCTs, published between 2020 and 2025, involved a total of 459 participants (40 of whom were lost to follow-up), with ages ranging from 16 to 30 years. Participants were allocated to either the MOPs group or the traditional orthodontic (non-MOPs) group (Tables 1, 2, and Supplementary Table S1) (16–31). Eight of the studies were designed as split-mouth RCTs (16, 22, 23, 25–28, 31). Dentofacial malocclusion types included Class I bimaxillary protrusion, Class II division 1 malocclusion, anterior crowding, and canine retraction. Various bracket systems (e.g., SPEED, MBT, Edgewise), archwire types (e.g., TMA, stainless steel), and auxiliary anchorage designs (e.g., TADs, mini-implants, Nance-TPA) were used, with applied forces ranging from 120 to 250 g. Types of tooth movement included canine retraction, anterior en-masse retraction, crowding alleviation in both arches, and incisor retraction.

**Table 1:** Characteristics of the involved studies.

Author/year	Sample size (n), M/F (n), and age (mean ± SD)	Malocclusion	Bracket system, ARCH wire Anchorage Force system	Type of tooth movement	MOP protocol	MOP Treatment Location	Conclusions
Li (2022)	20, 9/11, 16.8	NR	SPEED 0.022″ 0.019 × 0.025″ TMA; Nance-TPA; 150 g coil springs on power arms	Canine retraction	Propel, 2 perforations in same site 5 mm in depth. The first-time application is at the start of retraction, and the second time is approximately 12 weeks apart from the first-time application.	Maxillary The middle root region on the distal aspect of the maxillary canine.	This 12-week randomized split-mouth controlled clinical trial showed two MOPs that are 5 mm deep, applied once prior to space closure, did not create clinically significant increase in maxillary premolar space closure.
Shahrin (2021)	30,5/25, 22.66 ± 3.27	Moderate crowding of the upper labial segment	MBT 0.022 × 0.028″, 0.014 NiTi, NR	The initial orthodontic alignment phase of maxillary anterior crowding	Propel, 1 perforation distal to the canines, each perforation was 1.5 mm wide and 3 mm deep. MOPs were repeated at every four-week intervals visit until completion of the alignment stage.	Maxillary Between the anterior teeth from the upper right canine to the upper left canine, except in the midline alveolar bone.	Acceleration of orthodontic tooth movement with adjunctive MOPs therapy during the alignment phase does not exacerbate EARR in patients with moderate crowding of the upper labial segment in comparison with controls.
Chandorikar (2022)	52, NR, 21.35 ± 2.2	Class 1 bi-dentoalvelar protrusion	MBT 0.018″; 0.016 × 0.022″ SS; FavAnchor™SAS, 1.6 × 8 mm; 9 mm Niti closed coil spring with a force of 250 g per side was used for retraction	Anterior teeth retraction	Mini-implant (FavAnchor™SAS, India) of 1.6 × 8 mm size. 3 perforations were made at equidistance in each interradicular alveolar bone of all six anterior teeth, each perforation was 1.6 mm wide and 5 mm deep. One-time application at the start of retraction.	Maxillary&Mandibular In each interradicular alveolar bone of all six maxillary anterior teeth.	Within the settings of the current RCT, en-masse retraction when combined with single application of micro-osteoperforations did not pose an increased risk of root resorption or alveolar bone changes compared to routine sliding mechanics.
Kilinc (2023)	30, 13/17, 17.51 ± 2.79	NR	G&H Orthodontics, 0.014-inch NiTi, NR	The tooth movement of the mandibular anterior teeth	Propel, 3 perforations were created with a disposable device, each 1.5 mm in diameter and 3 mm in depth. They were placed vertically at equal intervals, with the first 2 mm apical to the alveolar crest and the remaining two 1 mm apart. After bonding and arch wire placement, MOPs were repeated every four weeks at each visit.	Mandibular Interproximally between the mandibular canines, lateral incisors, and central incisors.	The leveling of mandibular anterior teeth was accelerated by piezocision over 16 weeks, predominantly in the first 12 weeks, whereas MOP had no effect. It was concluded that piezocision is an acceptable procedure and has no destructive effect on the periodontal tissue.
Mordente (2024)	37, 17/20, 23.25 ± 6.46	Maxillary anterior region mild to moderate crowding	Edgewise 0.022″; 0.017 × 0.025″ SS; TADs 1.5 mm width, 6 mm length; 200 g Closed NiTi springs	Maxillary incisors’ retraction	Propel, MOPs were performed with a 1.6-mm diameter stainless steel surgical bur. Two 3 mm deep on the buccal surface and one 5 mm deep on the palatal region. All MOPs were performed only once and on the same day that the maxillary incisors’ retraction was begun.	Maxillary In maxillary anterior teeth. The first MOP was performed 6 mm away from the gingival margin, and the second was performed 5 mm from the first.	MOPs did not accelerate the retraction of the maxillary incisors, nor were they associated with greater incisor inclination or root resorption.
Raghav (2022)	60, 33/27, 20.52 ± 2.43	Class 1 bimaxillary protrusion or Class 2 div 1 malocclusion	MBT 0.018″ 0.016 × 0.022SS; mini-implants: 200gm NiTi closed coil spring of length 9 mm	Canine retraction	Micromotor, 3 perforations were made at equal distances distal to the canine in the extraction area and 2 perforations were made mesial to the canine roots bilaterally, each perforation was 2 mm wide and 5 mm deep. One-time application at the start of retraction.	Maxillary Between the maxillary canine and lateral incisor on both sides and in the extraction space of maxillary first premolar on both sides.	Micro-osteoperforations (MOPs) did not accelerate the rate of anterior en-masse retraction over a period of 4 months; however, it temporarily increases the rate of retraction only for first month and no affect on molar anchorage.
Gümüş (2023)	20, 13/7, 16.5 ± 2.3	Angle Class 2 div 1 malocclusion	MMS, 0.017 × 0.025″ SS, Mini screws, NiTi closed coil springs as 120 g	Canine retraction	Mini screws, 1.5 mm width, 8 mm length, with a screwdriver at three points distal to the canine. The Mini screw was inserted 3–4 mm deep. First time application at the start of retraction, the second time, 28 days after.	Maxillary In the center of the extraction region and 5, 8 and 11 mm away from the top of the alveolar crest.	MOPs did not significantly affect the rate of orthodontic tooth movement during canine retraction.
Singh (2023)	20, NR, 18–30	Class 2 div 1 malocclusion	MBT, 0.022 slot, 0.019 × 0.025 SS, TADs, 150 g NiTi closed coil springs	Canine retraction	Mini-implant, each perforation was 1.5 mm wide and 6 mm deep. 3 perforations were created on the buccal surface of the alveolar process on the experimental side. One-time application at the start of retraction	Maxillary The perforations were placed between the canine and second premolar at the extraction site, vertically 3 mm apart, with the first perforation 5 mm from the free gingival margin.	The study recommends that MOP procedure has substantial potential to be used as an adjunct to the routine mechanotherapy for accelerating tooth movement, as it may reduce treatment time by half in the first four weeks after the MOP procedure.
Sahin (2023)	28, 12/26, 16.25	Mandibular crowding	MMS, 0.022× 0.028, 0.016 × 0.022 Niti, NR	The phase of alignment	Propel, the device includes a 1.5 mm thick perforation tip and depth setting (3 mm, 5 mm, 7 mm). MOPs was performed only once at day 1.	Mandibular MOPs were suggested to be 3 mm deep in anterior region, 5 mm deep in premolar region, and 7 mm deep in the posterior region in the alveolar bone.	Alignment stage was shortened with MOP application. There was no difference between groups for patient satisfaction and pain level except for the first appointment. No difference was observed between the groups regarding cephalometric values. Clinically insignificant inflammation was observed in periodontal tissues for both groups.
Golshah (2021)	25, 14/11, 16–25	Class 2 div 1 malocclusion	Roth 22 band, 0.017 × 0.025-inch NiTi wires, G2 miniscrews, NiTi closed coil springs (150 g）	Canine retraction	G2 miniscrews, 5 perforations (3 in the buccal plate and 2 in the palatal plate) were created in the MOP side. The holes had an internal diameter of 1.6 mm and depth of 3–4 mm. One-time application before canine retraction.	Maxillary In buccal side, the first perforation had 3 mm distance from the distal surface of the canine and 6 mm distance from the gingival margin. In palatal side, the first perforation had 3 mm distance from the distal surface of the canine and 7 mm distance from the gingival margin.	MOP could not accelerate canine retraction but decreased the degree of canine tipping.
Thomas (2021)	33, 24/9, 22.1 ± 2.19	Class 1 bimaxillary protrusion Class 2 div 1 malocclusion	MBT 0.018, 0.016 × 0.022 SS, Micro-implants, NiTi closed coil springs (150 g）	After 6 months of extraction of premolars and micro-implant placement	Osteem Implant Corporation, 3 perforations were placed 3 mm apart vertically on the mesial and distal aspect of the canine root, starting at a point 6 mm apical from the alveolar crest with 2 mm width and 4 mm depth. One-time application before canine retraction.	Maxillary Placed 5 mm apical to the alveolar crest between the maxillary second premolar and first molar on both sides.	An increase in the rate of tooth movement can be achieved without any periodontal adverse effects in the first 45 days of the MOP procedure. The effectiveness of the MOP procedure on the rate of tooth movement gradually declined thereafter.
Yadav (2025)	20, 9/11, 19.70 ± 2.38	Angle Class 1 malocclusion with bimaxillary protrusion/proclined anterior teeth.	MBT 0.022 × 0.028, 0.019 × 0.025” SS, NR, 150 g 9 mm closed coil NiTi Spring	Canine retraction	Propel, 3 vertical perforations (1.5 mm diameter, 5 mm depth on the intervention side, for a total of six applications throughout the canine retraction period. MOPs were repeated every 28 days, for a total of six applications throughout the canine retraction period (7.6 ± 1.2 months).	Maxillary&Mandibular respectively 2–3 mm distal to the canine root.	MOP effectively accelerates OTM but increases the risk of root and alveolar bone resorption. The associated reduction in IL-4 may contribute to enhanced osteoclastic activity. Clinical application should be case-dependent, with caution in patients at risk of periodontal or root compromise.
Gulduren (2020)	18, 11/7, 19.94 ± 2.06	Bilateral Class II molar relationship	NR, Intraoral mini-screw, 500 g NiTi coil springs	Distalization initiated	Mini-screw, 2 perforations were performed at a depth of 5 to 6 mm, Once a week for a total of three times.	Maxillary Between the 2nd premolars and first molars, first molars and 2nd molars and distal to the 2nd molars	A 1.17-fold increase in the rate of tooth movement in the MOP group compared with the contralateral side was recorded. However, the accelerating effect of MOPs was lower than expected. The mean pain level was statistically greater in the MOP group compared to the contralateral side only on the first day of application.
Kumar (2024)	20, 7/13, 19.90 ± 2.43	NR	MBT 0.022 × 0.028, 0.019 × 0.025 SS, TADS, Niti closed-coil springs with a standard force of 150 g	The phase of alignment	FavAnchor, diameter 1.5 mm; length 4 mm, 3 vertical perforations in the labial cortical plate along the long axis of the six anterior teeth in each interdental region and 3 perforations distal to the root of the canines on both sides. One-time application at the beginning of space closure.	Maxillary&Mandibular respectively 1st perforations were located 6 mm from the free gingival margin; 2nd MOP was marked 5 mm from the first one in the apical/cervical direction; 3rd the third MOP was marked 5 mm above the 2nd MOP.	The use of MOPs is effective in increasing the rate of en masse tooth retraction in both the maxillary and the mandibular arch. The rate of tooth movement was greater even in the post-MOP period as compared to the control group.
Fattori (2020)	18, 7/11, 24.1 ± 6	Severe class 3	SLB Roth 0.022″ 019 × 25″ SS Mini-screws 9 mm Niti closed coil spring (200 g）	Anterior teeth retraction	Propel, 3 vertical perforations in the midway space between canine and 2nd premolar, 6–8 mm deep. Repeated after every activation session until space closure was completed.	Maxillary&Mandibular Three vertical perforations were in the extraction space between the canine and the second premolar.	3 MOPs were inefficient for accelerating tooth movement during anterior retraction. MOPs produced more impact on OHRQOL immediately following the MOP procedure and after 3 days
Babanouri (2020)	28, 7/5, 26.1 ± 9.1	Class 2 div 1	MBT 0.022-inch, 0.016 × 0.022″ SS wire mini-screw NiTi closed coil spring 150 g	Canine retraction	Mini-screws with 1.2 mm diameter. 3 perforations with to a depth of 1 mm, between the distal of the canine and the mesial of the 2nd premolar. The first MOP was located 5 mm away from the free gingival margin. One-time application at the start of retraction.	Maxillary Starting 5 mm apical to the free gingival margin as the site of the first perforation, additional perforations were made vertically at 3-mm intervals, so that the vertical distance between adjacent perforations was 3 mm.	MOPs were effective in accelerating tooth movement over a period of 3 months, but not clinically significant. There was no increase in the level of pain and discomfort due to MOPs.

NR, No report; div, division; mm, millimeter; MBT, McLaughlin, Bennett, and Trevisi brackets; MMS, Mini Master Series; NiTi, Nickel-titanium; SS, stainless steel; TADs, Temporary anchorage devices; OTM, Orthodontic tooth movement.

**Table 2:** Tooth movement and root resorption of the involved studies.

Author/year	Total participants(n/n) Group	Follow- up	Outcomes (primary/secondary, measurements)	Root Resorption (mm)	Tooth Movement	
					Distance (mm)	Rate (mm/month)
Li (2022)	17 (17/17) T: MOPs C: Non-MOPs, Traditional orthodontics	12weeks (4w, 8w, 12w)	1. Amount of tooth movement; 2. Pain levels (VAS). Distance between the contact points of the canine and second premolar was measured from the casts using digital calipers.	NR	T: 3.52 ± 0.98 C: 2.84 ± 0.84	T: 1.17 ± 0.33^a^ C: 0.95 ± 0.28^a^
Shahrin (2021)	28 (14/14) T: MOPs C: Non-MOPs, Traditional orthodontics	6 months (0 m, 6 m)	1. Root resorption. Crown length was measured from the mid-incisal edge to the midpoint between the mesial and distal CEJ. Root length was measured from this CEJ midpoint to the root apex. Apical root resorption was defined as the millimetric difference in root length between T1 and T2.	T: 0.13 ± 0.84 (*P* = 0.238) C: 0.14 ± 1.07 (*P* = 0.225)	NR	NR
Chandorikar (2022)	44 (22/22) T: MOPs C: Non-MOPs, Traditional orthodontics	6 months (0 m, 6 m)	1. Root resorption; 2. Alveolar bone thickness and height. Root resorption was calculated by subtracting T2 measurements from T1 measurements. The root length was measured as the perpendicular distance from the CEJ line to the root apex with the virtual measurement tools on CBCT images.	Maxillary: T: 0.79 ± 0.29 C: 0.73 ± 0.20 Mandibular: T: 0.71 ± 0.36 C: 0.70 ± 0.24	NR	NR
Kilinc (2023)	29 (14/15) T: MOPs C: Non-MOPs, Traditional orthodontics	16 weeks (4w, 8w, 12w, 16w)	1. Tooth movement; 2. Periodontal parameters (PI, GI, PD); 3. OHIP-14; 4. Pain (VAS). For each cast model, contact point deviation was measured linearly using digital calipers. The amount of irregularity and the quantitative index results were also obtained for each model. The model measurements were made using a digital caliper by a blind researcher (B.K.), and all the measurements were taken twice by the same researcher at 1-week intervals.	NR	T: 6.97 ± 1.73 C: 5.95 ± 1.28	T: 1.74 ± 0.43^a^ C: 1.49 ± 0.32^a^
Mordente (2024)	37 (18/19) T: MOPs C: Non-MOPs, Traditional orthodontics	4 months (14d, 2 m, 3 m, 4 m)	1. The rate of tooth movement; 2. Anchorage loss; 3. Root length changes in the maxillary central incisors. Maxillary incisor displacement was calculated as the change in position from T0 to T1–T5, expressed as the mean movement of the four incisors per patient. Root resorption was assessed by comparing initial and final central incisor lengths (incisal edge to apex), with any decrease indicating apical shortening.	T: 0.83 ± 2.18^a^ C: 0.76 ± 2.97^a^	T: 2.18 ± 0.60^a^ C:1.84 ± 0.88^a^	T: 0.55 ± 0.15^a^ C: 0.46 ± 0.22^a^
Raghav (2022)	55 (28/27) T: MOPs C: Non-MOPs, Traditional orthodontics	4 months (1 m, 2 m, 3 m, 4 m)	1. The rate of enmasse retraction; 2. Anchorage loss. On both sides, the perpendicular distance from the mesial surface of the maxillary first molar to the antero-medial surface of the third palatal ruga was measured, and changes from T0 to each follow-up were used to calculate molar mesial movement (anchorage loss) in mm/month.	NR	T: 2.13 ± 0.39^a^ C:1.74 ± 0.35^a^	T: 0.53 ± 0.12^a^ C: 0.44 ± 0.01^a^
Gümüş (2023)	20 (20/20) T: MOPs C: Non-MOPs, Traditional orthodontics	3 months (NR)	1. Amount of tooth movement. 2. The rate of tooth movement. The distance from the most distal point of the maxillary canine to the most mesial point of the second premolar was measured at baseline and again after 3 months of retraction; their difference represented 3-month canine distalization.	NR	T: 2.88 ± 1.32 C: 2.51 ± 1.41	T: 0.96 ± 0.76^a^ C: 0.84 ± 0.81^a^
Singh (2023)	20 (20/20) T: MOPs C: Non-MOPs, Traditional orthodontics	56 days (28d, 56d)	1. Amount of tooth movement. 2. The rate of tooth movement. 3. Pain (VAS). Alginate impressions were taken at T0 (before retraction), T1 (after 28 days), and T2 (after 56 days) to measure the amount of canine movement on the dental models.	NR	T: 1.59 ± 0.22^a^ C:0.83 ± 0.38^a^	T: 0.80 ± 0.36^a^ C: 0.42 ± 0.57^a^
Sahin (2023)	28 (14/14) T: MOPs C: Non-MOPs, Traditional orthodontics	4 months (NR)	1. Rate of mandibular tooth alignment. 2. Cephalometric variables. 3. Periodontal parameters. 4. Pain (VAS). Plaster models were converted to digital models using a scanning device. A software program was used to perform dental arch measurements on digital models.	NR	T: 4.27 ± 1.75^a^ C:2.82 ± 0.87^a^	T: 1.21 ± 0.50^a^ C: 0.62 ± 0.19^a^
Golshah (2021)	25 (25/25) T: MOPs C: Non-MOPs, Traditional orthodontics	3 months (1 m, 2 m, 3 m)	1. Rate of canine movement. 2. Amount of orthodontic tooth movement. A baseline digital model was obtained, and the three-dimensional distance between the same distal prominence point of the canine on the baseline and at the current follow-up time point was measured.	NR	T: 6.10 ± 1.37 C: 5.40 ± 1.27	T: 1.22 ± 0.27^a^ C: 1.08 ± 0.25^a^
Thomas (2021)	30 (30/30) T: MOPs C: Non-MOPs, Traditional orthodontics	NR	1. Amount of tooth movement. 2. Anchorage loss. 3. Periodontal parameters. 4. Alveolar bone level 5. Root length. Canine movement was measured with a Vernier/digital caliper as the change in distance from a fixed reference point. The linear distance from the micro-implant head to the first molar tube hook was recorded every 15 days. Root length was defined as the perpendicular distance between a CEJ reference line and a parallel line through the root apex.	T: 0.24 ± 1.41# C: 0.30 ± 1.27^a^	T: 3.61 ± 0.8 C: 2.82 ± 0.6	T: 1.20 ± 0.27^a^ C: 0.94 ± 0.20^a^
Yadav (2025)	20 (20/20) T: MOPs C: Non-MOPs, Traditional orthodontics	90 days (NR)	1. Root resorption. 2. Canine angulation. 3. Alveolar bone level and thickness. 4. Rate of canine retraction. 5. IL-4 levels in GCF. On CBCT images, root length (mm) was measured along the tooth's long axis from the CEJ to the apex at T0 and T1, and the angular change between T0 and T1 indicated canine tipping/uprighting. The CEJ–alveolar crest distance (mesial and distal) was recorded to assess crestal bone height changes. Plaster models were scanned to obtain STL files, which were analyzed digitally; changes in the distance from the canine cusp tip to a reference line were used to calculate total retraction and the mean monthly canine movement.	Maxillary T: 1.30 ± 0.41 C: 0.80 ± 0.11 Mandibular T: 1.24 ± 0.19 C: 0.85 ± 0.12	Maxillary^a^ T: 9.73 ± 1.29 C: 5.85 ± 2.13 Mandibular^a^ T: 10.34 ± 1.22 C: 5.17 ± 1.06	Maxillary T: 1.28 ± 0.17 C: 0.77 ± 0.28 Mandibular T: 1.36 ± 0.16 C: 0.68 ± 0.14
Gulduren (2020)	18 (9/9) T: MOPs C: Non-MOPs, Traditional orthodontics	84 days (28d, 56d, 84d)	1. Amount of tooth movement. 2. Periodontal parameters. 3. Pain (VAS). Using 3D models, maxillary molar displacement was measured and compared on the MOP side, the contralateral side, and in the control group at 3, 6, 9, and 12 weeks;	NR	T: 2.42 ± 0.43^a^ C:2.34 ± 0.15^a^	T:0.67 ± 0.03^a^ C:0.61 ± 0.03^a^
Kumar (2024)	20 (10/10) T: MOPs C: Non-MOPs, Traditional orthodontics	7.6 ± 1.2 months (28d, 56d, 84d, 112d, 140d, 168d)	1. The rate of tooth movement; 2. Pain (VAS). Plaster casts were scanned to construct 3D digital models. In the maxillary arch, a mid-palatal reference line was drawn, from which perpendicular lines were constructed tangent to the distal surface of the canines and the mesial surface of the second premolars. These reference lines were used to define the extraction spaces and to measure the amount of space closure, thereby determining the distance and rate of anterior tooth retraction.	NR	Maxillary:^a^ T: 4.24 ± 1.36 C: 2.76 ± 0.88 Mandibular:^a^ T: 3.68 ± 1.56 C: 2.64 ± 1.04	Maxillary:^a^ T: 1.06 ± 0.34 C: 0.69 ± 0.22 Mandibular:^a^ T = 0.92 ± 0.39 C = 0.66 ± 0.26
Fattori (2020)	18 (9/9) T: MOPs C: Non-MOPs, Traditional orthodontics	12 weeks (3w, 6w, 9w, 12 w)	1. Rate of Tooth Movement; 2. Space Closure Duration; 3. Oral Health Impact Profile-14. Distance from the most distal point in the canine and most mesial point in the 2nd premolar. Closing time between groups (mm/ month). Quality of life assessment	NR	T: 5.38 ± 0.50^a^ C:4.91 ± 0.49^a^	T: 0.67 ± 0.18^a^ C: 0.61 ± 0.17^a^
Babanouri (2020)	25 (12/13) T: MOPs C: Non-MOPs, Traditional orthodontics	4 months (1 m, 2 m, 3 m, 4 m)	1. Rate of canine retraction; 2. Pain (VAS). Cementoenamel junction and marginal bone crest from the buccal and lingual sides; numeric scale for pain intensity the distance between the canine and lateral incisor measured at three points (incisal, middle, and cervical). The amount of pain associated to MOP was evaluated in the day of canine retraction and 24 h later.	NR	T: 2.45 ± 0.20^a^ C:1.93 ± 0.13^a^	T: 0.76 ± 0.173^a^ C: 0.62 ± 0.11^a^

a There is no direct data in the article, calculated by SPSS based on the data already provided in the article; VAS: Visual analog scores; OHIP-14: Oral Health Impact Profile-14; NR: no report; T: Treatment group (MOPs); C: Control group (Non-MOPs, Traditional orthodontics);.

In these 16 studies (16–31), MOPs were performed in either the maxilla or mandible depending on the teeth involved and the study design. The number of perforations ranged from 1 to 5, with depths of 3–7 mm and widths of 1.5–2 mm. Follow-up duration ranged from 1 to 7.6 months. MOPs were applied multiple times in 8 RCTs, repeated at four-week intervals or at subsequent visits. Primary and secondary outcomes, measurement methods, and conclusions are also detailed in Tables 1, 2.

### 3.2 Risk of bias and applicability concerns

**Figure 2:**
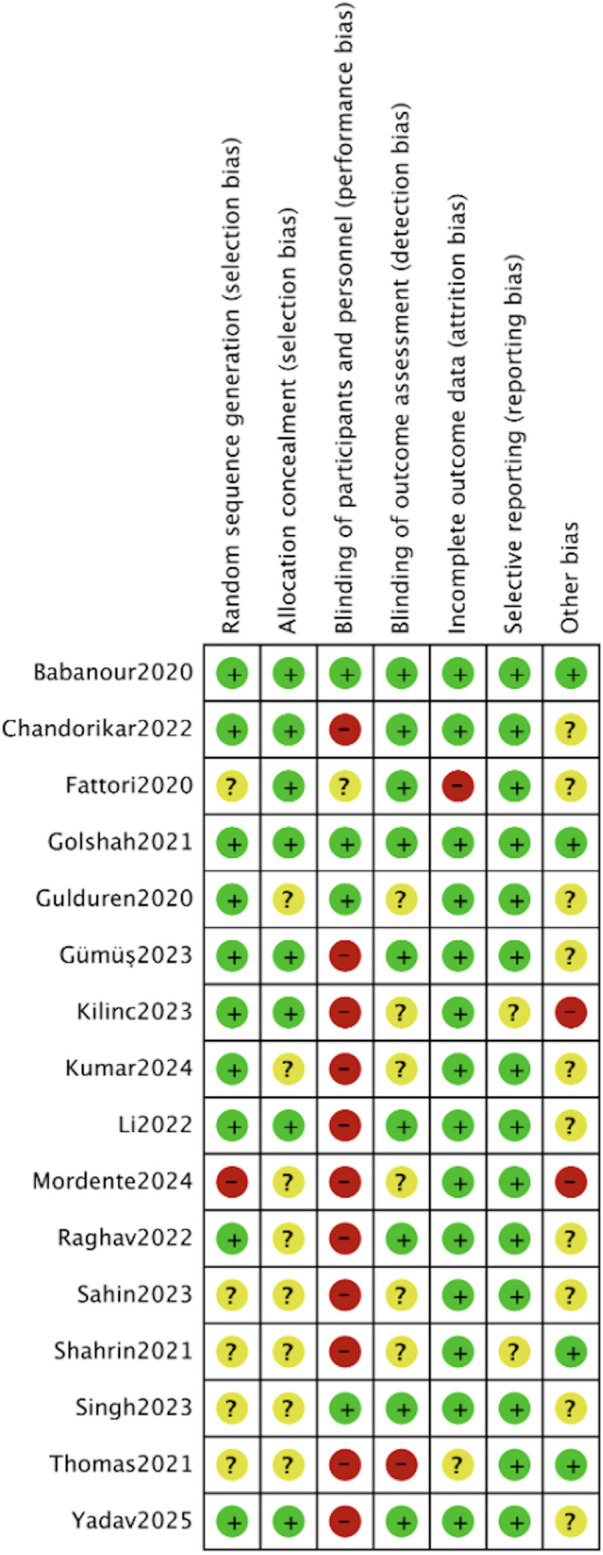
Risk of bias summary plot for all included RCTs, assessed via the cochrane risk of bias 2 tool. Each row represents one included study, and each column represents a bias domain.

**Figure 3:**
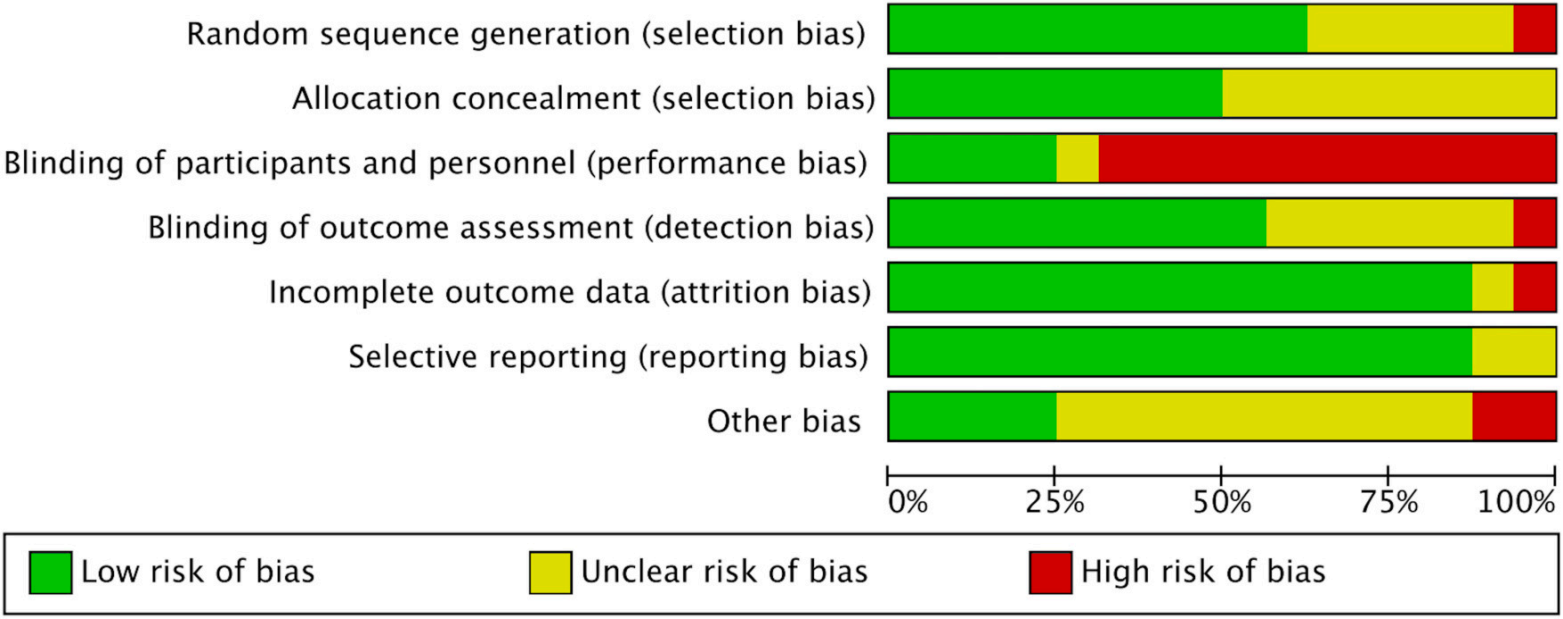
Risk of bias overall summary plot across all included studies. This plot summarizes the proportion of studies with low risk of bias, some concerns, and high risk of bias for each of the 5 bias domains.

The risk of bias of the included studies was evaluated using the Cochrane Risk of Bias tool (ROB 2), which assesses potential bias across several domains, including randomization, intervention implementation, outcome data completeness, outcome measurement, and selective reporting. The overall risk of bias is summarized in Figures 2, 3.

Most studies applied appropriate randomization methods, such as computer-generated random number tables, randomization software, or split-mouth designs, with allocation concealment achieved through sealed opaque envelopes, thereby minimizing selection bias. Examples include Li (16), Shahrin (17), Chandorikar (18), Raghav (21), Singh (23), Sahin (24), Golshah (25), Thomas (26), Yadav (27), Gulduren (28), and Fattori (30), all of which employed rigorous randomization and allocation protocols. However, a few studies reported limitations: Kilinc (19) had small and uneven group sizes with unclear baseline comparability; Mordente (20) included two non-random allocations due to anatomical considerations; and Gulduren (28) provided no further details to confirm whether appropriate randomization tools and allocation concealment methods were used. Overall, the risk of bias arising from the randomization process was generally low across studies.

Given the nature of surgical interventions such as micro-osteoperforation, blinding of participants and operators was not feasible in any of the studies, leading to a moderate risk of performance bias. For instance, Shahrin (17), Chandorikar (18), Kilinc (19), Raghav (21), and Golshah (25) explicitly acknowledged the lack of participant and operator blinding. Nevertheless, most studies implemented standardized protocols conducted by a single experienced orthodontist, which helped to minimize variability (Sahin (24), Thomas (26).

Most studies had good follow-up rates with minimal attrition. Li (16), Shahrin (17), Raghav (21), Sahin (24), Thomas (26), Yadav (27), and Kumar (29) reported either complete datasets or negligible dropout rates. Chandorikar (18) reported minor loss to follow-up (8/60), and Kilinc (19) excluded a single participant, while Mordente (20) reported protocol deviations and occasional dropouts. Importantly, several studies applied transparent reporting or intention-to-treat analyses (e.g., Mordente (20), minimizing attrition bias overall.

Most studies employed objective and reliable outcome measurement methods, such as cone-beam computed tomography (CBCT), digital model superimposition, or calibrated clinical tools. Several included blinded outcome assessors (Li (16), Chandorikar (18), Raghav (21), Sahin (24), Golshah (25), Thomas (26), Yadav (27), and Kumar (29), which reduced detection bias. Nevertheless, studies that included subjective outcomes such as pain assessments were more vulnerable to measurement bias, particularly when assessor blinding was not specified (Shahrin (17), Chandorikar (18), and Kilinc (19). Overall, the risk of detection bias was generally low to moderate.

Most studies fully reported both primary and secondary outcomes without evidence of selective reporting. Pre-registration in trial registries further enhanced transparency in several studies (e.g., Sahin (24), Thomas (26). However, some studies lacked trial registration or prespecified analysis plans, leaving reporting bias unclear or at moderate risk.

In summary, the included studies exhibited an overall low to moderate risk of bias: the risk of bias in randomization, outcome data completeness, and selective reporting was generally low across all studies, while moderate risks of performance and detection bias were consistently present due to the unblind nature of surgical interventions. These potential sources of bias should be carefully considered when interpreting the overall findings of this meta-analysis.

### 3.3 Primary outcomes

**Figure 4:**
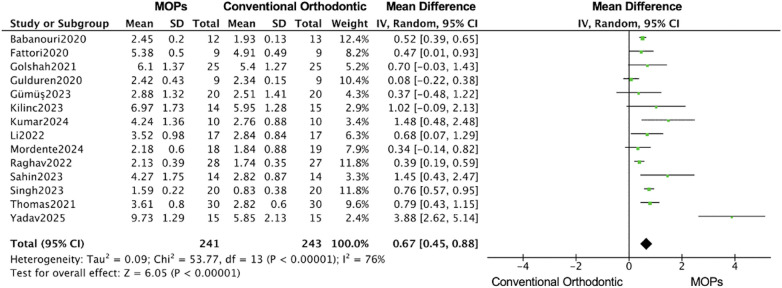
Forest plot of the meta-analysis evaluating the effect of micro-osteoperforation (MOPs) on total orthodontic tooth movement distance. This plot compares the intervention group receiving MOPs adjunctive to orthodontic treatment vs. the control group receiving conventional orthodontic treatment alone. The pooled mean difference (MD) with 95% confidence intervals (CI) was calculated using a random-effects model to account for between-study heterogeneity.

**Figure 5:**
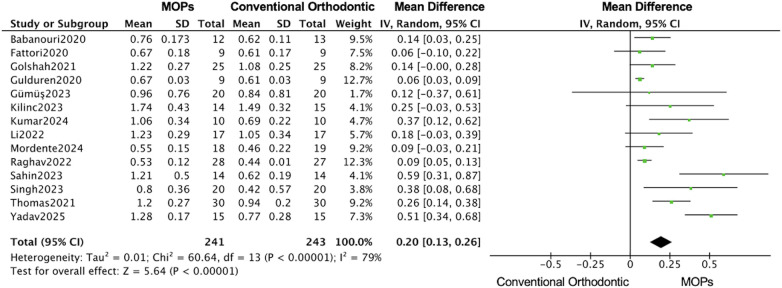
Forest plot of the meta-analysis evaluating the effect of micro-osteoperforation (MOPs) on monthly orthodontic tooth movement rate. This plot compares the intervention group receiving MOPs adjunctive to orthodontic treatment vs. the control group receiving conventional orthodontic treatment alone. The pooled mean difference (MD) with 95% confidence intervals (CI) was calculated using a random-effects model.

A total of 14 RCTs reported the primary outcomes of tooth movement distance and rate. For secondary outcomes, root resorption was assessed in 4 RCTs, anchorage loss in 3 RCTs, periodontal parameters in 4 RCTs, and patient-reported outcomes (including pain and quality of life) in 8 RCTs. 11 studies focused on maxillary (16, 19–31), 7 studies used Propel instrument (16, 19, 20, 24, 27, 30, 31) and 7 studies performed multiple MOPs (21–23, 25, 26, 28, 29). Overall, in this meta-analysis, the MOPs group achieved an increase of 0.67 mm (95%CI:0.45–0.88) in the distance of tooth movement (Figure 4), and a monthly increase of 0.20 mm (95%CI:0.13–0.26) in the rate of tooth movement (Figure 5), when compared with traditional orthodontic treatment. Moreover, in Kilinic's research (19), the difference in tooth movements improvement within the first month between MOPs and piezocision was minimal without significance (piezocision:7.60 ± 1.55 mm, MOPs:6.97 ± 1.73 mm). Total 9 studies demonstrate a significant impact of MOPs on the distance of tooth movement (16, 21, 23, 25–27, 29–31). 6 studies failed to demonstrate a significant impact of MOPs on the rate of tooth movement (16, 19, 20, 22, 25, 28). The variability in results may be attributed to factors such as treatment location, frequency of MOPs, and perforation technique instruments.

**Figure 6:**
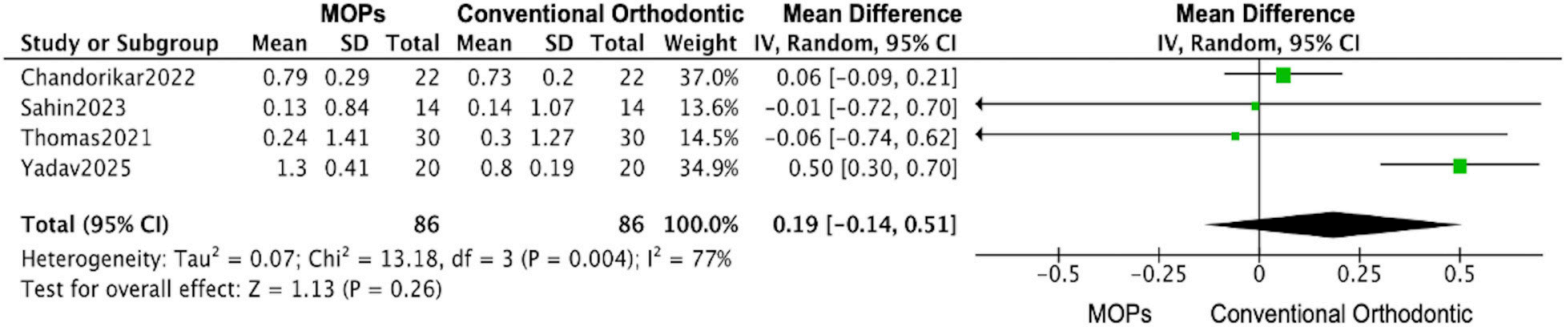
Forest plot of the meta-analysis evaluating the effect of micro-osteoperforation (MOPs) on root resorption. This plot compares changes in root length between the MOPs intervention group and the conventional orthodontic treatment control group. The pooled mean difference (MD) with 95% confidence intervals (CI) was calculated using a random-effects model.

**Figure 7:**
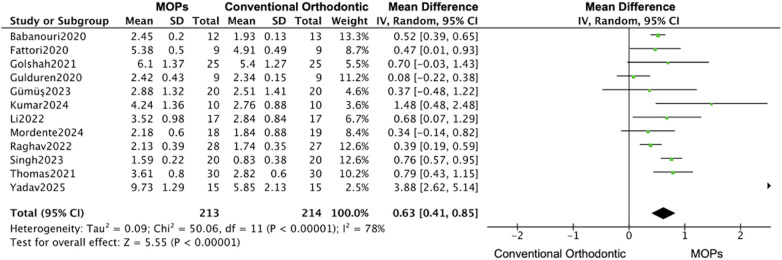
Subgroup forest plot of total orthodontic tooth movement distance, restricted to maxillary teeth. This analysis compares the effect of MOPs vs. conventional orthodontic treatment for studies focusing on maxillary sites. The pooled mean difference (MD) with 95% confidence intervals (CI) was calculated using a random-effects model.

**Figure 8:**
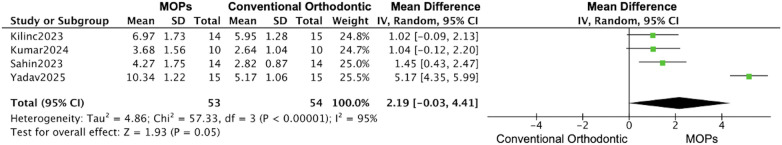
Subgroup forest plot of total orthodontic tooth movement distance, restricted to mandibular teeth. This analysis compares the effect of MOPs vs. conventional orthodontic treatment for studies focusing on mandibular sites. The pooled mean difference (MD) with 95% confidence intervals (CI) was calculated using a random-effects model.

**Figure 9:**
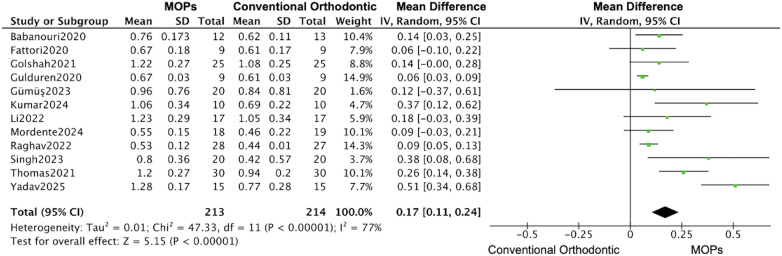
Subgroup forest plot of monthly orthodontic tooth movement rate, restricted to maxillary teeth. This analysis compares the effect of MOPs vs. conventional orthodontic treatment for studies focusing on maxillary sites. The pooled mean difference (MD) with 95% confidence intervals (CI) was calculated using a random-effects model.

**Figure 10:**
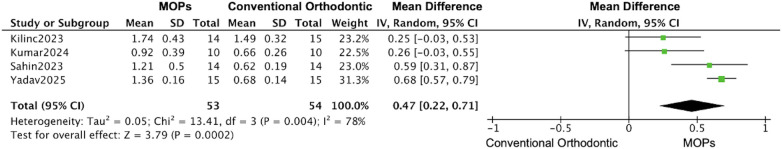
Subgroup forest plot of monthly orthodontic tooth movement rate, restricted to mandibular teeth. This analysis compares the effect of MOPs vs. conventional orthodontic treatment for studies focusing on mandibular sites. The pooled mean difference (MD) with 95% confidence intervals (CI) was calculated using a random-effects model.

The subgroups analysis based on the perforation location (maxillary or mandibular) revealed that the MOPs group increased 0.63 mm (95% CI: 0.41–0.85; 95% PI: 0.18–1.08) distance of tooth movement in maxillary (16, 20–23, 25–31), while 2.19 mm (95% CI: −0.03–4.41; 95% PI: −1.25–5.63) in mandibular (Figures 7, 8) (19, 24, 27, 29), and a monthly rate increase of 0.17 mm (95% CI: 0.11–0.24; 95% PI: 0.05–0.29) in maxillary (16, 20–23, 25–31), while 0.47 mm (95% CI: 0.22–0.71; 95% PI: 0.10–0.84) in mandibular (Figures 9, 10) (19, 24, 27, 29).

The subgroups analysis based on different instrument (propel or others) revealed that the MOPs group increased 1.21 mm (95% CI: 0.54–1.87; 95% PI: 0.32–2.10) distance of tooth movement in using propel (16, 19, 20, 24, 27, 30, 31), while 0.55 mm (95% CI: 0.29–0.80; 95% PI: 0.08–1.02) in using other instrument (Supplementary Figure S6) (21–23, 25, 26, 28, 29), and a monthly rate increase of 0.26 mm (95% CI: 0.09–0.43; 95% PI: 0.01–0.51) in using propel (16, 19, 20, 24, 27, 30, 31), while 0.14 mm (95% CI: 0.07–0.20; 95% PI: 0.03–0.25) in using other instrument (Supplementary Figure S6) (21–25, 28, 29).

**Figure 11:**
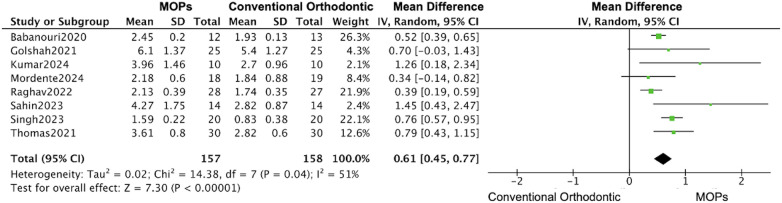
Subgroup forest plot of total orthodontic tooth movement distance, restricted to studies using one-time MOPs application. This analysis compares the effect of one-time MOPs vs. conventional orthodontic treatment. The pooled mean difference (MD) with 95% confidence intervals (CI) was calculated using a random-effects model.

**Figure 12:**
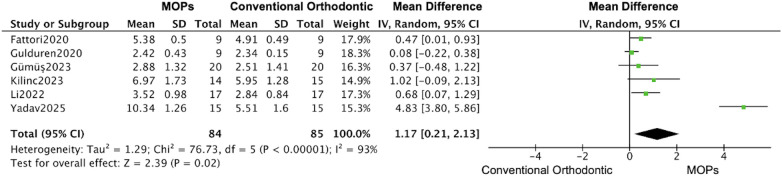
Subgroup forest plot of total orthodontic tooth movement distance, restricted to studies using multiple MOPs application. This analysis compares the effect of multiple MOPs vs. conventional orthodontic treatment. The pooled mean difference (MD) with 95% confidence intervals (CI) was calculated using a random-effects model.

**Figure 13:**
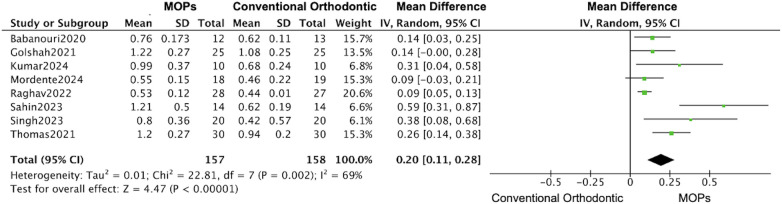
Subgroup forest plot of monthly orthodontic tooth movement rate, restricted to studies using one-time MOPs application. This analysis compares the effect of one-time MOPs vs. conventional orthodontic treatment. The pooled mean difference (MD) with 95% confidence intervals (CI) was calculated using a random-effects model.

**Figure 14:**
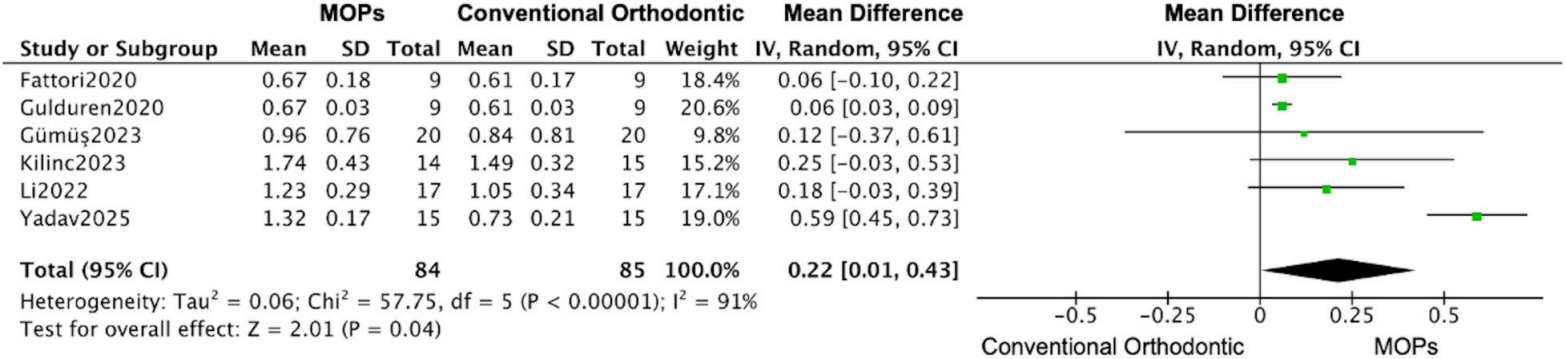
Subgroup forest plot of monthly orthodontic tooth movement rate, restricted to studies using multiple/repeated MOPs application. This analysis compares the effect of multiple MOPs vs. conventional orthodontic treatment. The pooled mean difference (MD) with 95% confidence intervals (CI) was calculated using a random-effects model.

The subgroups analysis based on the perforation frequency (one or multiple MOPs) revealed that an increase of 0.61 mm (95% CI: 0.45–0.77; 95% PI: 0.38–0.84) (20, 21, 23–26, 29, 31) in the distance of tooth movement was calculated based on the date form one-time MOPs studies, while the large increase was observed in multiple MOPs studies (1.17 mm, 95% CI: 0.21–2.13; 95% PI: –0.35–2.69) (Figures 11, 12) (16, 19, 22, 27, 28, 30), and a similar enhancement of monthly rate was demonstrated in both subgroups (single MOPs: 0.20 mm, 95% CI: 0.11–0.28; 95% PI: 0.06–0.34 vs. multiple MOPs: 0.22 mm, 95% CI: 0.01–0.43; 95% PI: –0.15–0.59) (Figures 13, 14) (16, 19, 22, 27, 28, 30).

The studies with one-time MOPs on maxillary or mandibular revealed that the increase of distance (max:0.62 mm, 95%CI:0.45–0.79, vs. mand:0.60 mm, 95%CI:0.44–0.76) (Supplementary Figure S5) and rate (max:0.20 mm, 95%CI:0.12–0.29, vs. mand:0.19 mm, 95%CI:0.11–0.28) (Supplementary Figure S5) of tooth movement was similar (20–22, 24–26, 29, 31). The studies with multiple MOPs on maxillary or mandibular revealed that the increase of rate of tooth movement was similar (max:0.20 mm, 95%CI:0.03–0.36, vs. mand:0.23 mm, 95%CI:-0.04–0.50) (Supplementary Figure S5), while the increase of tooth movement distance was higher in mandibular (max:0.91 mm, 95%CI:0.23–1.59, vs. mand:1.28 mm, 95%CI:0.06–2.50) (Supplementary Figure S5) (16, 19, 22, 27, 28, 30).

Meta-regression was performed to identify the key factors contributing to the high heterogeneity (I^2^ = 76% for total movement distance, I^2^ = 79% for monthly movement rate) (32). The results revealed that perforation frequency (*P* = 0.023) and instrument type (*P* = 0.018) were significant predictors of movement distance heterogeneity, explaining 31.2% and 27.8% of the variance, respectively. Specifically, multiple MOPs applications and the use of the Propel device were associated with larger acceleration effects, which aligned with the subgroup analysis findings. In contrast, patient age (*P* = 0.346), tooth type (*P* = 0.412), and perforation depth (*P* = 0.289) did not significantly contribute to heterogeneity. For the monthly movement rate, no single covariate reached statistical significance (all *P* > 0.05), suggesting that other unmeasured factors (e.g., applied orthodontic force magnitude, patient oral hygiene status) may also influence the observed variability. These results highlight that standardizing perforation frequency and instrument type may help reduce heterogeneity in future MOPs studies (32).

### 3.4 Secondary outcomes

All secondary outcomes were assessed to evaluate the safety of MOPs, including root resorption, anchorage loss, periodontal tissue status, and patient-reported outcomes (pain and quality of life).

4 RCTs assessed root resorption outcomes (17, 18, 26, 27). 2 of these found a significant association between MOPs and increased root resorption (17, 27), while the remaining 2 did not observe a notable effect (18, 26). This meta-analysis revealed that, compared with conventional orthodontic treatment, the MOPs group showed an average increase of 0.19 mm (95% CI: −0.14 to 0.51) in root resorption (Figure 6), and the data was similar in both maxillary or mandibular (max: 0.18 mm, 95%CI:-0.13–0.50, vs. mand:0.15 mm, 95%CI:-0.15–0.44) (Supplementary Figure S4).

Regarding anchorage loss, Mordente quantified the mesial movement of the molars using 3D digital models(UR6T: 0.04 ± 0.43 C:−0.16 ± 0.69, UL6T: 0.08 ± 0.33 C:−0.06 ± 0.49), Raghav (T: 0.19 mm/month C: 0.23 mm/month) and Thomas (T: 0.05 ± 1.63 mm C: 0.06 ± 1.56 mm) use the amount of molar movement representing anchorage loss, all of them found no significant differences between the experimental and control groups (Table 3) (20, 21, 26).

**Table 3:** Other related scores of the involved studies.

Author/year	OHIP	Pain	Anchorage [methods, results (mm)]	Periodontal Parameter (mm)
Li (2022)	NR	Pain levels (VAS) 0d: T: 12.78 ± 17.08 C: 9.17 ± 18.01 1d: T: 20.56 ± 23.69 C: 19.44 ± 24.61 2d: T: 10 ± 14.04 C: 9.72 ± 13.45 7d: T: 3.89 ± 6.98 C: 3.06 ± 5.72	NR	NR
Chandorikar (2022)	NR	NR	NR	NR
Kilinc (2023)	OHIP-14 T: 31.00 ± 11.18 C: 30.20 ± 5.95	Pain (VAS) 1d: T: 67.65 ± 22.17 C: 42.05 ± 8.25 4d: T: 21.98 ± 25.48 C: 24.82 ± 20.74 7d: T: 4.61 ± 6.21 C: 5.82 ± 6.52	NR	PI: T: 0.46 ± 0.57 C: 0.44 ± 0.55 GI: T: 0.72 ± 0.4° C: 0.42 ± 0.50 MI: T: 0.58 ± 0.31 C: 0.37 ± 0.30 PD: T: 0.50 ± 0.21 C: 0.03 ± 0.27
Mordente (2024)	NR	NR	Quantify the mesial movement of the molars using 3D digital models. UR6T: 0.04 ± 0.43 C:−0.16 ± 0.69 UL6 T: 0.08 ± 0.33 C:−0.06 ± 0.49	NR
Raghav (2022)	NR	NR	Digital models were obtained, a reference plane was established, and the amount of molar movement was calculated. T: 0.19 mm/month C: 0.23 mm/month	NR
Singh (2023)	NR	Pain (VAS) 1h: T: 3.10 ± 1.6° C: 2.00 ± 1.94 24h: T: 1.10 ± 0.88 C: 0.80 ± 0.92 72h: T: 0.0° C: 0.10 ± 0.32	NR	NR
Sahin (2023)	NR	Pain (VAS) T0: T: 5.96 ± 2.93 C: 2.64 ± 1.98. T2: T: 4.25 ± 2.01 C: 6.25 ± 2.33	NR	GI: T: 0.44 ± 0.08, C: 0.54 ± 0.11 PI: T: 0.04 ± 0.08 C:0.08 ± 0.07 ## BOP: T: 12.76 ± 4.95 C: 18.37 ± 8.30 GT: T: 0.64 ± 0.18 C: 0.89 ± 0.17
Thomas (2021)	NR	NR	The distance representing the “micro-implant–molar tube relationship” was designated as D2 and was used to monitor mesial movement of the molar, that is anchorage loss. T: 0.05 ± 1.63 mm C: 0.06 ± 1.56 mm	1d: PD: T: 1.31 ± 0.4 C: 1.26 ± 0.3 RAL: T: 8.34 ± 1.1 C: 8.28 ± 1.1 90d: PD: T: 1.72 ± 0.4 C: 1.47 ± 0.3 RAL:T: 8.34 ± 1.1 C: 8.28 ± 1.1
Gulduren (2020)	VAS Discomfort score; T0: T: 3.333 ± 3.123 C: 4.556 ± 2.920 T1: T: 2.333 ± 2.693 C: 2.056 ± 1.286 Eating difficulty score; T0: T: 3.333 ± 3.123 C: 7.278 ± 2.138 T1: T: 2.333 ± 2.693 C: 3.056 ± 2.007 Speech problems score: T0: T: 6.667 ± 3.162 C: 4.333 ± 2.398 T1: T: 1.333 ± 2.18° C: 1.556 ± 2.128	Pain (VAS) T0: T: 5.444 ± 2.963 C: 3.833 ± 3.437 T1: T: 2.333 ± 1.732 C: 2.222 ± 2.694	NR	PI:# 1st premolar T: 0.023 ± 0.473 C: 0.174 ± 0.638 1st molar T: 0.394 ± 0.405 C: 0.452 ± 0.484 2nd molar T: 0.243 ± 0.553 C: 0.445 ± 0.503 GI:# 2nd premolar T: 0.389 ± 0.342 C: 0.163 ± 0.236 1st molar T: 0.768 ± 0.426 C: 0.442 ± 0.346 2nd molar T: 0.574 ± 0.391 C: 0.127 ± 0.319 PD:# 2nd premolar T: 0.214 ± 0.348 C: 0.024 ± 0.274 1st molar T: 0.389 ± 0.415 C: 0.308 ± 0.295 2nd molar T: 0.227 ± 0.379 C: 0.005 ± 0.243 BOP:# 2nd premolar T: 0.088 ± 0.146 C: 0.125 ± 0.096 1st molar T: 0.015 ± 0.192 C: 0.366 ± 0.168 2nd molar T: 0.259 ± 0.177 C: 0.164 ± 0.118
Kumar (2024)	NR	Pain (VAS) 1d: T: 5.0 ± 1.24 7d: T: 2.10 ± 0.99 14d: T: 0.8 ± 1.13	NR	NR
Fattori (2020)	OHIP-14 Functional limitation: 4h: T: 0.80 ± 0.92 C: 0.58 ± 1.73 72h: T: 1.50 ± 2.12 C: 0.33 ± 1.15 Psychological discomfort 4h: T: 4.90 ± 2.38 C: 1.00 ± 1.28 72h: T: 3.30 ± 2.79 C: 1.00 ± 1.28 Physical disability 4h: T: 4.40 ± 2.8° C: 1.83 ± 1.53 72h: T: 4.50 ± 2.88 C: 1.17 ± 1.75 Psychological disability: 4h: T: 2.20 ± 1.32 C: 0.50 ± 0.67 72h: T: 2.70 ± 2.16 C: 0.17 ± 0.39 Social disability 4h: T: 2.70 ± 1.89 C: 0.92 ± 1.44 72h: T: 3.00 ± 2.54 C: 0.92 ± 1.16 Physical disability 4h: T: 3.10 ± 2.38 C: 3.00 ± 2.62 72h: T: 0.92 ± 1.38 C: 0.42 ± 0.90)	OHIP-14 4h: T: 5.20 ± 2.15 C: 4.67 ± 2.31 72h: T: 0.70 ± 1.89 C: 3.42 ± 2.23	NR	NR
Babanouri (2020）	NR	Pain (VAS) 0h: T: 3.66 ± 2.34 C: 2.96 ± 2.26. 24h: T:1.75 ± 2.22 C: 1.40 ± 1.50	NR	NR

VAS, Visual analog scores; OHIP-14, Oral Health Impact Profile-14; NR, no report; T, Treatment group; C, Control group; GI, Gingival index; PI, Periodontal index; MI, Mobility; PD, Periodontal Pocket Depth; RAL, Relative attachment level; GT, Gingival thickness.

Periodontal parameters were reported in 4 studies (Table 3) (19, 24, 26, 28). In Kilinc's research (19), PI was similar in both groups, while GI (T: 0.72 ± 0.4° C: 0.42 ± 0.50), mobility (T: 0.58 ± 0.31 C: 0.37 ± 0.30) and PD (T: 0.50 ± 0.21 C: 0.03 ± 0.27) were higher in MOPs group. Sahin's results demonstrated similar PI and GI, and the BOP (T: 12.76 ± 4.95 C: 18.37 ± 8.30) and GT (T: 0.64 ± 0.18 C: 0.89 ± 0.17) were lower in MOPs group (24). Thomas also reported no significance between two groups in PD and RAL (26).

8 RCTs revealed pain scores and only 3 RCTs reported the life quality (16, 19, 23, 24, 28–31). 7 studies employed the Visual Analogue Scale (VAS) to quantify patients’ postoperative pain and 1 study used the OHIP-14 questionnaire (16, 19, 23, 24, 28–31). The experimental data indicated that the pain score of the experimental group (MOPs group) on the day of surgery was significantly higher than that of the control group (no MOPs). The difference in pain between the experimental group and the control group gradually decreased at subsequent assessment time points, and no significant difference was observed. Both VAS [Gulduren] and the OHIP-14 questionnaire [Kilinc and Fattori] were used to evaluate patients’ quality of life following surgery (19, 28, 30). Kilinc did not find significance between two group after MOPs surgery (19). The results of Fattori and Gulduren indicated that the impact of surgery on daily life peaked within the first three days postoperatively but gradually diminished as time progressed (28, 30).

## 4 Discussion

The GRADE assessment revealed that the certainty of evidence for the primary outcomes (tooth movement distance and rate) was moderate, mainly due to moderate heterogeneity and performance/detection bias in the included studies. For secondary outcomes, the certainty of evidence was low to very low: low for root resorption and anchorage loss (caused by limited number of included studies and imprecision of data), and very low for periodontal parameters and pain/quality of life (due to inconsistent measurement methods and subjective outcome assessment). This evidence grade should be considered when interpreting the findings and translating them into clinical practice.

To better contextualize the contribution of the present study, we compared it with three key previous meta-analyses focusing on MOPs for orthodontic tooth movement (6 33, 34). First, compared with Sivarajan et al. (33), which included 10 RCTs (*n* = 326) and focused primarily on maxillary tooth movement, our study expanded the sample size to 16 RCTs (*n* = 459) and included 4 RCTs focusing on mandibular MOPs, revealing a more pronounced acceleration effect in the mandible (2.19 mm increase in movement distance vs. 0.63 mm in the maxilla). This finding refines the understanding of MOPs' site-specific efficacy, which was not fully addressed in earlier work. Second, Alferm et al. (34) reported a monthly tooth movement rate increase of 0.15 mm with MOPs but did not explore subgroup differences based on instrument type or perforation frequency. Our subgroup analysis demonstrated that the Propel device (0.26 mm/month) yielded a 85.7% higher acceleration rate than other instruments (0.14 mm/month), and multiple perforations doubled the total movement distance (1.17 mm vs. 0.61 mm for single perforations), providing actionable clinical parameters for optimal MOPs application. Third, Al-Khalifa & Baeshen (6) conducted a systematic review (not a meta-analysis) and highlighted the lack of long-term safety data, but our study comprehensively synthesized secondary outcomes (root resorption, periodontal parameters, pain) from 4 to 8 RCTs each, confirming that MOPs does not cause clinically significant adverse effects. Additionally, our study included 8 RCTs published between 2020 and 2025 (vs. no post-2020 studies in the three previous works), ensuring the timeliness and generalizability of the findings. Collectively, these updates address the limitations of prior literature and provide more precise, patient-centered evidence for clinical decision-making.

This meta-analysis on MOPs suggests that, although findings remain heterogeneous, MOPs promotes short-term acceleration of orthodontic tooth movement under controlled conditions. However, its long-term efficacy remains inconclusive and warrants further investigation. Overall, based on 16 studies, the MOPs group achieved an increase of 0.67 mm (95% CI: 0.45–0.88) in the total distance of tooth movement and a monthly increase of 0.20 mm (95% CI: 0.13–0.26) in the rate of tooth movement compared with traditional orthodontic treatment. These findings are consistent with those of previous studies, among which nine demonstrated a significant increase in movement distance and eight reported a significant increase in movement rate (Figures 4, 5) (16, 19–31).

Specifically, six included studies (22, 27–31) revealed that MOPs significantly accelerated the rate of space closure during the first month, but this effect was not sustained in later stages, suggesting that MOPs may confer only a brief accelerating effect during the early phase of tooth movement. In contrast, four other studies (20, 23, 24, 26) showed that MOPs facilitated orthodontic gap closure within three months; however, the clinical significance was minimal and not statistically significant—a finding consistent with previous meta-analyses (35–37).

Given the high heterogeneity among studies—12 focused on the maxilla (16, 20–23, 25–31), 7 used the Propel device (16, 19, 20, 24, 27, 30, 31), and 6 applied multiple MOPs (16, 19, 22, 27, 28, 30)—we further categorized the included studies by surgical site (maxilla vs. mandible). Interestingly, we observed that following MOPs surgery, both the increase in movement distance (maxilla: 0.63 mm [95% CI: 0.41–0.85]; mandible: 2.19 mm [95% CI: −0.03 to 4.41]) and the monthly rate of tooth movement (maxilla: 0.17 mm [95% CI: 0.11–0.24]; mandible: 0.47 mm [95% CI: 0.22–0.71]) were greater in the mandible than in the maxilla. This suggests that MOPs may more effectively accelerate tooth movement when applied for mandibular space closure. However, this finding should be interpreted with caution, as only four RCTs involved the mandible (19, 24, 27, 29), and only two of these reported statistically significant results (24, 27).

In line with the considerations of many researchers and clinicians, we also recognize that the frequency of MOPs procedures may be a significant factor affecting the distance and speed of tooth movement. Subgroup analysis indicated that multiple perforations significantly enhanced the total distance of tooth movement (single MOPs: 0.61 mm [95% CI: 0.45–0.77]; multiple MOPs: 1.17 mm [95% CI: 0.21–2.13]), whereas the monthly rate of tooth movement remained largely unchanged at approximately 0.2 mm. This may be attributable to the fact that the accelerating effect of MOPs occurs predominantly within the first month (26, 27). According to some studies, increasing the number or depth of perforations may enhance local stimulation, thereby generating sustained bone remodeling and accelerating tooth movement during orthodontic treatment. However, all 14 studies included in this meta-analysis involving tooth movement employed more than one perforation; thus, further research is needed to determine the optimal frequency, quantity, and depth of perforations.

It is worth noting that our findings differ slightly from those of previous meta-analyses. We observed that the use of the standard Propel device for MOPs resulted in greater movement distance (1.21 mm [95% CI: 0.54–1.87] vs. 0.55 mm [95% CI: −0.29 to 0.80]) and rate (0.26 mm [95% CI: 0.09–0.43] vs. 0.14 mm [95% CI: 0.07–0.20]). Nevertheless, this result should be adopted with caution due to the relatively high heterogeneity among studies. Therefore, promoting standardized surgical protocols and the use of standardized instruments in future MOPs treatments may contribute to more stable acceleration effects.

Some studies, including those by Mordente, Yadav, and Kumar, have also reported that although MOPs facilitated the movement of certain root segments, the overall rate of tooth movement did not significantly improve (20, 27, 29), indicating that the effects of MOPs on root movement are localized and transient.

Anchorage changes remain a critical focus in orthodontic treatment. Current evidence suggests that MOPs have a limited clinical impact on orthodontic anchorage; however, further high-quality clinical investigations are necessary given the sparse evidence and heterogeneous anchorage assessment methods. In this meta-analysis, three studies reported anchorage changes, measured as molar displacement (i.e., anchorage loss), and observed a slight, non-significant decrease (20, 21, 26). The primary mechanism of MOPs is to reduce bone density around the surgical site, while bone density surrounding the anchorage teeth remains unchanged. Moreover, insufficient evidence exists to support the effects of repeated perforations or perforation depth on anchorage during orthodontic treatment, warranting further investigation.

Root resorption does not appear to be clinically significantly exacerbated by MOPs, despite their potential to accelerate short-term tooth movement. In this study, root resorption was observed in some cases (17, 18, 20); however, the extent was generally mild and clinically acceptable compared with traditional orthodontics. Previous animal studies have also demonstrated that MOPs increase the total number of osteoclasts over a short duration but have a relatively minor impact on root resorption (38, 39). It is the long-term presence of osteoclasts that leads to external root resorption, rather than a transient increase in osteoclast numbers. Histologically, root resorption of the first premolar, which was subjected to orthodontic force and extracted at 28 days, increased by 42% in the MOPs group (37, 40). Nevertheless, this finding should be interpreted with caution, as the short study duration differs substantially from full-length orthodontic treatment in real-world patients. In addition, no studies have reported direct damage to tooth roots during the drilling procedure—a potential risk that warrants serious consideration.

MOPs are safe and exert minimal effects on periodontal tissues, without causing significant periodontal damage. Kilinc et al. clearly demonstrated that over a 16-week follow-up period, no significant changes in periodontal parameters were observed (19). Sahin et al. (24) provided a detailed assessment of gingival index (GI), plaque index (PI), bleeding on probing (BOP), and gingival thickness (GT), reporting that although GI and PI increased slightly during treatment, the changes were within clinically acceptable ranges and not statistically significant. Thomas et al. found no significant differences in attachment loss (AL), while probing depth (PD) increased slightly likely attributable to mechanical forces from orthodontic traction (26). Furthermore, at the gene expression level, MOPs do not significantly affect the periodontal ligament (PDL) gene expression profile during orthodontic treatment (38, 41). Therefore, MOPs do not compromise periodontal health. In future MOPs treatments, promoting standardized surgical procedures and clearly defining optimal drilling positions, depths, and quantities may help mitigate potential threats to periodontal health.

MOPs slightly exacerbate patients' subjective experiences, such as pain and postoperative quality of life, in the short term compared with traditional orthodontic treatment. Both Gulduren et al. and Kumar et al. reported that pain scores in the experimental group were significantly higher on the day of the procedure, with differences between groups gradually diminishing over time (28, 29). According to studies by Shahrin, Kilinc, and Sahin et al. (17, 19, 24), pain following MOPs is relatively pronounced in the early postoperative period and progressively decreases over time. Patients' quality of life was assessed using the visual analog scale (VAS) (28) or the OHIP-14 questionnaire (19, 30) following surgery. Kilinc et al. found that quality of life was not significantly affected by MOPs, while other studies reported impacts only within the first three postoperative days, which gradually resolved (19, 30). These findings indicate that MOPs is a relatively mild intervention for accelerating orthodontic movement and is generally well accepted by patients. Consistent with the low to very low GRADE evidence for secondary outcomes, the findings on root resorption and periodontal parameters across included studies were heterogeneous, yet no clinically significant adverse effects were observed in any of the assessed safety outcomes. Collectively, multiple studies consistently demonstrated that MOPs, as a minimally invasive orthodontic acceleration technique, had a mild and safe impact on root resorption, periodontal tissues, and quality of life, with acceptable levels of pain. Compared with three key previous meta-analyses (6 33, 34), our study's expanded sample size, updated RCT inclusion, site/instrument/frequency-specific subgroup analyses, and comprehensive safety outcome synthesis enhance the evidence base for MOPs application. Moreover, the clinical application of MOPs necessitates a nuanced evaluation of its effects on tooth movement, tailored to diverse treatment scenarios and individual patient characteristics.

## 5 Limitations

This review was limited to English-language publications, potentially excluding relevant studies in other languages. Long-term evaluations are necessary to assess adverse effects of MOPs, as none were reported in the included studies. Further research is also needed on perforation depth and frequency, as well as postoperative pain and swelling. Outcome heterogeneity may stem from displacement of reference points; future studies should adopt fixed markers (e.g., mini-implants) to improve measurement reliability. While current MOPs research primarily focuses on tooth movement and root resorption, periodontal outcomes warrant greater attention. Well-designed, long-term trials are essential to confirm the clinical benefits of MOPs.

## 6 Conclusion

Based on the findings of this exploratory systematic review and meta-analysis, MOPs demonstrate potential in accelerating tooth movement during orthodontic treatment, particularly within the first three months after surgery. Although MOPs may enhance short-term tooth movement, their long-term efficacy remains uncertain, and outcomes vary across studies. The acceleration effect, while present, tends to be localized and of limited clinical significance. Importantly, MOPs do not appear to significantly increase the risk of root resorption; most studies report only mild and clinically manageable effects. Similarly, periodontal health is largely unaffected, with no notable increases in gingival inflammation or bone loss observed. However, due to the invasive nature of MOPs and potential side effects such as discomfort, patient compliance may be compromised. Therefore, the use of MOPs should be carefully evaluated and is best reserved for complex or treatment-resistant tooth movements, cases in which conventional methods have proven insufficient, or situations in which faster tooth movement is prioritized over patient comfort.

## Data Availability

The original contributions presented in the study are included in the article/Supplementary Material, further inquiries can be directed to the corresponding author/s.

## References

[1] Kole H. Surgical operations on the alveolar ridge to correct occlusal abnormalities. Oral Surg Oral Med Oral Pathol. (1959) 12:515–29. 10.1016/0030-4220(59)90153-7 13644913

[2] Frost HM. The regional acceleratory phenomenon: a review. Henry Ford Hosp Med J. (1983) 31:3–9. 6345475

[3] Wilcko WM Wilcko T Bouquot JE Ferguson DJ. Rapid orthodontics with alveolar reshaping: two case reports of decrowding. Int J Periodon Restorat Dent. (2001) 21:9–19. 11829041

[4] Kim YS Kim SJ Yoon HJ Lee PJ Moon W Park YG. Effect of piezopuncture on tooth movement and bone remodeling in dogs. Am J Orthod Dentofacial Orthop. (2013) 144:23–31. 10.1016/j.ajodo.2013.01.022 23810042

[5] Feizbakhsh M Zandian D Heidarpour M Farhad SZ Fallahi HR. The use of micro-osteoperforation concept for accelerating differential tooth movement. J World Fed Orthod. (2018) 7:56–60. 10.1016/j.ejwf.2018.04.002

[6] Al-Khalifa KS Baeshen HA. Micro-osteoperforations and its effect on the rate of tooth movement: a systematic review. Eur J Dent. (2021) 15:158–67. 10.1055/s-0040-1713955 32610360 PMC7902111

[7] Sharma K Raghavan S Batra P. Osseous evidence behind micro-osteoperforation. Am J Orthod Dentofacial Orthop. (2021) 159:e81. 10.1016/j.ajodo.2020.11.008 33546836

[8] Teixeira CC Khoo E Tran J Chartres I Liu Y Thant LM Cytokine expression and accelerated tooth movement. J Dent Res. (2010) 89:1135–41. 10.1177/0022034510373764 20639508 PMC3318047

[9] Charavet C Lambert F Lecloux G Le Gall M. Accelerated orthodontic treatment using corticotomies: what are the minimally invasive alternatives? Orthodontie Française. (2019) 90:5–12. 10.1051/orthodfr/2019002 30994445

[10] Charavet C Lecloux G Bruwier A Vandenberghe B Le Gall M Lambert F. Selective piezocision-assisted orthodontic treatment combined with minimally invasive alveolar bone regeneration: a proof-of-concept. Int Orthod. (2018) 16:652–64. 10.1016/j.ortho.2018.09.021 30391131

[11] Gomes JRCL Vargas IA Rodrigues AFA Gertz LC Freitas MP Miguens SAQ Jr Micro-osteoperforation for enhancement of orthodontic movement: a mechanical analysis using the finite element method. PLoS One. (2024) 19:e0308739. 10.1371/journal.pone.0308739 39159186 PMC11332926

[12] Mosayebi N Khademi A Bagherieh S Abedi N Kargarfard M Tajmiri G The effect of micro-osteoperforation on root resorption, pulp vitality, and biological changes of teeth subjected to orthodontic tooth movement: a systematic review study. Dent Res J (Isfahan). (2023) 20:52. 10.4103/1735-3327.374809 37304419 PMC10247875

[13] Wagh SS Nehete A Gulve N Aher S Patil D Tambe M. Comparative evaluation of effect of micro-osteoperforation and mechanical vibration on rate of orthodontic tooth movement in young adults with bimaxillary protrusion. Cureus. (2023) 15:e36636. 10.7759/cureus.36636 37155450 PMC10122870

[14] Higgins JP Thompson SG Deeks JJ Altman DG. Measuring inconsistency in meta-analyses. Br Med J. (2003) 327(7414):557–60. 10.1136/bmj.327.7414.557 12958120 PMC192859

[15] Riley RD Higgins JP Deeks JJ. Interpretation of random effects meta-analyses. Br Med J. (2011) 342:d549. 10.1136/bmj.d549 21310794

[16] Li J Papadopoulou AK Gandedkar N Dalci K Darendeliler MA Dalci O. The effect of micro-osteoperforations on orthodontic space closure investigated over 12 weeks: a split-mouth, randomized controlled clinical trial. Eur J Orthod. (2022) 44:427–35. 10.1093/ejo/cjab079 35134142

[17] Shahrin AA Ghani SHA Norman NH. Effect of micro-osteoperforations on external apical root resorption: a randomized controlled trial. Korean J Orthod. (2021) 51:86–94. 10.4041/kjod.2021.51.2.86 33678624 PMC7940811

[18] Chandorikar H Bhad WA. Impact of micro-osteoperforations on root resorption and alveolar bone in en-masse retraction in young adults: a CBCT randomized controlled clinical trial. International Orthodontics. (2023) 21:100714. 10.1016/j.ortho.2022.100714 36502787

[19] Kilinc B Baka ZM. Comparison of the effectiveness of piezocision and micro-osteoperforation in leveling mandibular anterior teeth. Am J Orthod Dentofacial Orthop. (2023) 163:491–500. 10.1016/j.ajodo.2022.02.019 36517376

[20] Mordente CM Oliveira DD Palomo JM Cardoso PA Assis MAL Zenóbio EG The effect of micro-osteoperforations on the rate of maxillary incisors’ retraction in orthodontic space closure: a randomized controlled clinical trial. Prog Orthod. (2024) 25:6. 10.1186/s40510-023-00505-z 38342823 PMC10859353

[21] Raghav P Khera AK Bhasin P. Effect of micro-osteoperforations on rate of space closure by mini-implant supported maxillary anterior en-masse retraction: a randomized clinical trial. J Oral Biol Craniofac Res. (2021) 11:185–91. 10.1016/j.jobcr.2021.01.010 33598396 PMC7868723

[22] Bolat Gumus E Kinsiz E. Effects of miniscrew-facilitated micro-osteoperforations on the rate of orthodontic tooth movement: a split-mouth, randomized controlled trial. Journal of Orofacial Orthopedics. (2023) 84(Suppl 2):104–10. 10.1007/s00056-021-00371-6 35024875

[23] Singh S Jain AK Prasad RR Sahu A Priya P Kumari P. Effect of mini-implant assisted micro-osteoperforation on the rate of orthodontic tooth movement: a randomized clinical trial. J Orthod Sci. (2023) 12:62. 10.4103/jos.jos_18_23 38234639 PMC10793843

[24] Faik Sahin M Baysal A. The effect of micro-osteoperforation on the rate of tooth movement during the alignment stage in patients with mandibular crowding: a randomised controlled trial. Eur J Orthod. (2023) 45:505–16. 10.1093/ejo/cjad017 37167078

[25] Golshah A Moradi P Nikkerdar N. Efficacy of micro-osteoperforation of the alveolar bone by using mini-screw for acceleration of maxillary canine retraction in young adult orthodontic patients: a split-mouth randomized clinical trial. Int Orthod. (2021) 19:601–11. 10.1016/j.ortho.2021.09.006 34696998

[26] Thomas S Das SK Barik AK Raj SC Rajasekaran A Mishra M. Evaluation of physiodispenser-assisted micro-osteoperforation on the rate of tooth movement and associated periodontal tissue status during individual canine retraction in first premolar extraction cases: a split-mouth randomized controlled clinical trial. J World Fed Orthod. (2021) 10:89–97. 10.1016/j.ejwf.2021.05.001 34112627

[27] Yadav D Batra P Talwar A Sonar S Srivastava A. A comparative assessment of orthodontically induced root resorption and alveolar bone changes in adolescent orthodontic patients undergoing micro-osteoperforations-assisted canine retraction: a split-mouth randomized controlled trial. Clin Oral Investig. (2025) 29:429. 10.1007/s00784-025-06507-x 40888938

[28] Gulduren K Tumer H Oz U. Effects of micro-osteoperforations on intraoral miniscrew-anchored maxillary molar distalization: a randomized clinical trial. J Orofacial Orthop. (2020) 81:126–41. 10.1007/s00056-019-00207-4 32095922

[29] Kumar P Rampurawala AH Patil AS. Effect of micro-osteoperforations on the rate of en masse orthodontic tooth retraction: a randomized controlled trial. J Orofacial Orthop. (2024) 85:189–98. 10.1007/s00056-022-00420-8 36018346

[30] Fattori L Sendyk M de Paiva JB Normando D Neto JR. Micro-osteoperforation effectiveness on tooth movement rate and impact on oral health-related quality of life. Angle Orthod. (2020) 90:640–7. 10.2319/110819-707.1 33378487 PMC8032262

[31] Babanouri N Ajami S Salehi P. Effect of mini-screw-facilitated micro-osteoperforation on the rate of orthodontic tooth movement: a single-center, split-mouth, randomized, controlled trial. Prog Orthod. (2020) 21:7. 10.1186/s40510-020-00306-8 32147751 PMC7061095

[32] Thompson SG Sharp SJ. Explaining heterogeneity in meta-analysis: a comparison of methods. Stat Med. (1999) 18(20):2693–708. 10.1002/(SICI)1097-0258(19991030)18:20<2693::AID-SIM235>3.0.CO;2-V 10521860

[33] Sivarajan S Ringgingon LP Fayed MMS Wey MC. The effect of micro-osteoperforations on the rate of orthodontic tooth movement: a systematic review and meta-analysis. Am J Orthod Dentofacial Orthop. (2020) 157(3):290–304. 10.1016/j.ajodo.2019.10.009 32115107

[34] Alferm AB Elhousiny SA Alqahtani ND Alqahtani AM Alfaifi YA. Micro-osteoperforation and the rate of orthodontic tooth movement: systematic review and meta-analysis. Ann Med Health Sci Res. (2021) 11:16–20.

[35] Dos Santos CCO Mecenas P de Castro Aragón MLS Normando D. Effects of micro-osteoperforations performed with propel system on tooth movement, pain/quality of life, anchorage loss, and root resorption: a systematic review and meta-analysis. Prog Orthod. (2020) 21:27. 10.1186/s40510-020-00326-4 32715352 PMC7383046

[36] Inpanya P Chanmanee P Teerakanok S. Effects of micro-osteoperforation depths on canine retraction rate and root resorption: a systematic review and meta-analysis. Eur J Dent. (2025) 20. 10.1055/s-0045-1806932 PMC12494454 40315865

[37] Mheissen S Khan H Alsafadi AS Almuzian M. The effectiveness of surgical adjunctive procedures in the acceleration of orthodontic tooth movement: a systematic review of systematic reviews and meta-analysis. J Orthod. (2021) 48:156–71. 10.1177/1465312520988735 33546565

[38] Sugimori T Yamaguchi M Kikuta J Shimizu M Negishi S. Micro-osteoperforations accelerate tooth movement without exacerbating the progression of root resorption in rats. Biomolecules. (2024) 14:300. 10.3390/biom14030300 38540720 PMC10967735

[39] Kraiwattanapong K Samruajbenjakun B. Effects of different force magnitudes on corticotomy-assisted orthodontic tooth movement in rats. Angle Orthod. (2018) 88:632–7. 10.2319/103117-736.1 29714068 PMC8183137

[40] Sivarajan S Ringgingon LP Fayed MMS Wey MC Khoo EYH Alikhani M Physical properties of root cementum: part 26. Effects of micro-osteoperforations on orthodontic root resorption: a microcomputed tomography study. Am J Orthod Dentofacial Orthop. (2018) 153:204–13. 10.1016/j.ajodo.2017.05.036 29407497

[41] Spitz A Adesse D Gonzalez M Pellegrino R Hakonarson H Marañón-Vásquez GA Effect of micro-osteoperforations on the gene expression profile of the periodontal ligament of orthodontically moved human teeth. Clin Oral Investig. (2022) 26:1985–96. 10.1007/s00784-021-04178-y 34499218

