# Supplementary material for: Micro-osteoperforation for accelerating orthodontic tooth movement: a meta-analysis of short-term efficacy, safety, and optimal application parameters
Source: Front Dent Med. 2026 May 14;7:1814419. doi: 10.3389/fdmed.2026.1814419 (PMC13216033; doi:10.3389/fdmed.2026.1814419)
Supplement: Supplementary file 1 [file Supplementaryfile1.docx]

**Supplemental Material**

[Supplemental Method 1. Database Search Strategy and Results. 2](#_Toc176422142)

[Supplemental Method 2. PRISMA checklists. 4](#_Toc176422143)

[Supplemental Method 3. The study design, Randomization, Allocation concealment, Blinding, Loss and Analysis set of in the included studies 8](#_Toc176422145)

[Supplemental Figure 1. Meta-analysis of the distance of Tooth Movement# 13](#_Toc176422148)

[Supplemental Figure 2. Meta-analysis of the rate of Tooth Movement# 14](#_Toc176422149)

[Supplemental Figure 3. Meta-analysis of the root resorption# 15](#_Toc176422150)

[Supplemental Figure 4. Subgroup analyses of main outcomes of interest (based on Maxillary&Mandibular respectively ) 16](#_Toc176422151)

[Supplemental Figure 5. Subgroup analyses of outcomes of interest (based on one/multiple MOPs and Maxillary & Mandibular ) 21](#_Toc176422152)

[Supplemental Figure 6. Subgroup analyses of outcomes of interest (based on different instrument ) 26](#_Toc176422155)

[Supplemental Figure 7. Funnel plots and Egger’s test of outcomes of interest 29](#_Toc176422156)

**Supplemental Method 1. Database Search Strategy and Results**

| **Database** | **Search Strategy** | **Search Results** |
| --- | --- | --- |
| **PubMed** | ("orthodontic tooth movement" [Title/Abstract] OR "canine retraction"[Title/Abstract] OR "tooth movement [Title/Abstract] OR "teeth movement" [Title/Abstract] OR "root resorption" [Title/Abstract] OR "periodontal tissue"[Title/Abstract] OR "pain [Title/Abstract] OR osteocentesis [Title/Abstract] OR "Tooth Movement Techniques"[Mesh]) AND (micro-osteoperforation*[Title/Abstract] OR micro-perforation*[Title/Abstract] OR "flapless osteopuncture*" [Title/Abstract] OR MOP*[Title/Abstract] ) | 66 |
| **EMBASE** | ('orthodontic tooth movement':ti,ab OR 'canine retraction':ti,ab OR 'tooth movement':ti,ab OR 'teeth movement':ti,ab OR 'root resorption':ti,ab OR 'periodontal tissue':ti,ab OR pain:ti,ab OR osteocentesis:ti,ab OR 'tooth movement technique'/exp) AND (micro-osteoperforation*:ti,ab OR micro-perforation*:ti,ab OR 'flapless osteopuncture*':ti,ab OR MOP*:ti,ab) | 400 |
| **Scopus** | TITLE-ABS-KEY ("orthodontic tooth movement" OR "canine retraction" OR "tooth movement" OR "teeth movement" OR "root resorption" OR "periodontal tissue" OR "pain" OR "osteocentesis" OR "tooth movement techniques") AND TITLE-ABS-KEY ("micro-osteoperforation* OR micro-perforation* OR "flapless osteopuncture*" OR MOP*) | 149 |
| **Web of Science** | TS= ("orthodontic tooth movement" OR "canine retraction" OR "tooth movement" OR "teeth movement" OR "root resorption" OR "periodontal tissue" OR "pain" OR "osteocentesis" OR "Tooth Movement Techniques") AND TS= ("micro-osteoperforation" OR "micro-perforation" OR "flapless osteopuncture" OR MOP) | 78 |
| **Cochrane LIBRARY** | ("orthodontic tooth movement":ti,ab OR "canine retraction":ti,ab OR "tooth movement":ti,ab OR "teeth movement":ti,ab OR "root resorption":ti,ab OR "periodontal tissue":ti,ab OR "pain":ti,ab OR "osteocentesis":ti,ab OR [mh "Tooth Movement Techniques"]) AND (micro-osteoperforation:ti,ab,kw OR micro-perforation:ti,ab,kw OR "flapless osteopuncture":ti,ab,kw OR MOP:ti,ab,kw) | 120 |
| **LILACS** | db:LILACS AND (tw:"orthodontic tooth movement" OR tw:"canine retraction" OR tw:"tooth movement" OR tw:"teeth movement" OR tw:"root resorption" OR tw:"periodontal tissue" OR tw: "pain" OR tw: "osteocentesis" OR mh:"Tooth Movement Techniques") AND (tw: "micro-osteoperforation" OR tw: "micro-perforation" OR tw:"flapless osteopuncture" OR tw:MOP) | 134 |
| **Google Scholar** | ("orthodontic tooth movement" OR "canine retraction" OR "tooth movement" OR "teeth movement" OR "root resorption" OR "periodontal tissue" OR "pain" OR "osteocentesis" OR "tooth movement techniques") AND ("micro-osteoperforation" OR "micro osteoperforation" OR "micro-perforation" OR "micro perforation" OR "flapless osteopuncture" OR "micro-osteoperforation (MOP)") | 228 |

MEDLINE = Medical Literature Analysis and Retrieval System Online; EMBASE = Excerpta Medica Database; EMBASE = Excerpta Medica Database. LILACS: Latin American and Caribbean health sciences literature database;

**Supplemental Method 2. PRISMA checklists**

| **Section/topic** | **#** | **Checklist item** | **Reported on page #** |
| --- | --- | --- | --- |
| **TITLE** | | |  |
| Title | 1 | Identify the report as a systematic review, meta-analysis, or both. | 1 |
| **ABSTRACT** | | |  |
| Structured summary | 2 | Provide a structured summary including, as applicable: background; objectives; data sources; study eligibility criteria, participants, and interventions; study appraisal and synthesis methods; results; limitations; conclusions and implications of key findings; systematic review registration number. | 2 |
| **INTRODUCTION** | | |  |
| Rationale | 3 | Describe the rationale for the review in the context of what is already known. | 2 |
| Objectives | 4 | Provide an explicit statement of questions being addressed with reference to participants, interventions, comparisons, outcomes, and study design (PICOS). | 2 |
| **METHODS** | | |  |
| Protocol and registration | 5 | Indicate if a review protocol exists, if and where it can be accessed (e.g., Web address), and, if available, provide registration information including registration number. | 5 |
| Eligibility criteria | 6 | Specify study characteristics (e.g., PICOS, length of follow-up) and report characteristics (e.g., years considered, language, publication status) used as criteria for eligibility, giving rationale. | 5 |
| Information sources | 7 | Describe all information sources (e.g., databases with dates of coverage, contact with study authors to identify additional studies) in the search and date last searched. | 6 |
| Search | 8 | Present full electronic search strategy for at least one database, including any limits used, such that it could be repeated. | Supplemental Method 1 |
| Study selection | 9 | State the process for selecting studies (i.e., screening, eligibility, included in systematic review, and, if applicable, included in the meta-analysis). | 7 |
| Data collection process | 10 | Describe method of data extraction from reports (e.g., piloted forms, independently, in duplicate) and any processes for obtaining and confirming data from investigators. | 8 |
| Data items | 11 | List and define all variables for which data were sought (e.g., PICOS, funding sources) and any assumptions and simplifications made. | 6 |
| Risk of bias in individual studies | 12 | Describe methods used for assessing risk of bias of individual studies (including specification of whether this was done at the study or outcome level), and how this information is to be used in any data synthesis. | 8 |
| Summary measures | 13 | State the principal summary measures (e.g., risk ratio, difference in means). | 7 |
| Synthesis of results | 14 | Describe the methods of handling data and combining results of studies, if done, including measures of consistency (e.g., I^2^) for each meta-analysis. | 7 |

| **Section/topic** | **#** | **Checklist item** | **Reported on page #** |
| --- | --- | --- | --- |
| Risk of bias across studies | 15 | Specify any assessment of risk of bias that may affect the cumulative evidence (e.g., publication bias, selective reporting within studies). | 8,9; Figure 2,3 |
| Additional analyses | 16 | Describe methods of additional analyses (e.g., sensitivity or subgroup analyses, meta-regression), if done, indicating which were pre-specified. | 7 |
| **RESULTS** | | |  |
| Study selection | 17 | Give numbers of studies screened, assessed for eligibility, and included in the review, with reasons for exclusions at each stage, ideally with a flow diagram. | 9; Figure 1 |
| Study characteristics | 18 | For each study, present characteristics for which data were extracted (e.g., study size, PICOS, follow-up period) and provide the citations. | 9; Table 1. |
| Risk of bias within studies | 19 | Present data on risk of bias of each study | 9,10; Figure 2,3 |
| Results of individual studies | 20 | For all outcomes considered (benefits or harms), present, for each study: (a) simple summary data for each intervention group (b) effect estimates and confidence intervals, ideally with a forest plot. | 8-15 Figure 4-14; Supplemental Figure 1-7 |
| Synthesis of results | 21 | Present results of each meta-analysis done, including confidence intervals and measures of consistency. | 8-15 Figure 4-14; Supplemental Figure 1-7 |
| Risk of bias across studies | 22 | Present results of any assessment of risk of bias across studies. | 8-10 Supplemental Method 3 |
| Additional analysis | 23 | Give results of additional analyses, if done (e.g., sensitivity or subgroup analyses, meta-regression). | 8-15 Supplemental Figure 1-7 |
| **DISCUSSION** | | |  |
| Summary of evidence | 24 | Summarize the main findings including the strength of evidence for each main outcome; consider their relevance to key groups (e.g., healthcare providers, users, and policy makers). | 15 |
| Limitations | 25 | Discuss limitations at study and outcome level (e.g., risk of bias), and at review-level (e.g., incomplete retrieval of identified research, reporting bias). | 21 |
| Conclusions | 26 | Provide a general interpretation of the results in the context of other evidence, and implications for future research. | 22 |
| **FUNDING** | | |  |
| Funding | 27 | Describe sources of funding for the systematic review and other support (e.g., supply of data); role of funders for the systematic review. | 22-23 |

**Supplemental Method 3. The study design, Randomization, Allocation concealment, Blinding, Loss and Analysis set of in the included studies**

| **Author /year** | **Study design** | **Randomization** | **Allocation**  **concealment** | **Blinding** | **Loss** | **Analysis set** |
| --- | --- | --- | --- | --- | --- | --- |
| **Li (2020)** | two-arm parallel, split-mouth,  RCT | Randomization table by an investigator not involved in patient management. | Separate sealed and opaque envelopes was performed by a staff member not directly involved with the trial. | Participants and the operator was not blinded. The examiner was blinded. | 2 subjects were lost in both group in the analysis of pain scores.  No loss in tooth movement during T0-T2,3 subjects was not analyzed in tooth movement during T2-T3 because the space were closed at T2. | PPS |
| **Shahrin (2021)** | single-center, two-arm parallel, RCT## | The block randomization of six numbers (three odd and three even numbers). Odd numbers were allocated to the MOP group while even numbers were assigned to the control group. | Sequentially numbered opaque, sealed envelopes were used for concealed randomization. Cards were written with numbers and set in opaque sealed envelopes, which were kept by the central trial coordinator. | Participants and the operator was not blinded. The examiner was blinded. | 1 participant lost in MOPs group due to pregnant and 1participant lost in control group due to records were not collected at the right stage. | PPS |
| **Chandorikar (2022)** | single-center, two-arm parallel, RCT | The block randomization method using Cochran and Cox's (1957). 20 sets of random permutations of the first 16 integers. Another researcher from the institute carried out randomization. | Sealed envelopes were used for concealed randomization. | Participants and the operator was not blinded. The examiner was blinded. | 3 participants lost due to pandemic and 1lost due to discontinued intervention in both two groups. | PPS |
| **Kilinc (2023)** | RCT | NR | NR | Participants and the operator was not blinded. The examiner was blinded. | 1 participate lost in MOPs group | PPS |
| **Mordente (2024)** | RCT## | Randomization was performed using QuickCalcs software. | Concealing this allocation from the orthodontic team. The periodontist was informed of the participants’ allocation 1 week before the procedure. | Participants and the operator was not blinded. The examiner was blinded. | In MOPs group:1 participant lost due to irregular attendance and 2 participants were excluded due to digital models with collection problems. In control group:1 participant lost due to pregnant and 1 participant were excluded due to digital models with collection problems. | PPS |
| **Raghav (2022)** | two arm parallel, RCT## | Random allocation was done by block randomization method with a block size of 6. | The concealment was done in sequentially numbered, sealed, opaque envelopes which were shuffled by an independent investigator. | Participants and the operator was not blinded. The examiner was blinded. | 2 participants lost in MOPs group and 3 participants lost in control group. The reason was not reported. | PPS |
| **Gümüş (2023)** | split-mouth,  RCT | Randomization for either the right or left side was achieved the flip of the coin | NR | NR | No loss | FAS |
| **Singh (2023)** | single-center, split-mouth,  RCT | Randomization was performed using sealed, opaque envelopes containing the allocation codes. | Sealed envelopes were used for concealed randomization. | Triple blinding design was employed. MOPs were performed by the first author, while the treating orthodontist, outcome assessor, and statistician were all blinded to treatment allocation. | No loss | FAS |
| **Sahin (2023)** | two arm parallel,  RCT## | Random number table prepared using SPSS software (version 20.0; IBM) for the allocation of patients. | The allocation sequence was concealed using numbered and sealed opaque envelopes. | Blinding was used at the data collection and analysis stages | No loss | FAS |
| **Golshah (2021)** | split-mouth RCT## | Random Allocation Software (version 2.0) in 1:1 distribution and equal numbers. | Opaque sealed envelopes were all performed by one independent observer. | Blinding was used at the data collection and analysis stages | No loss | FAS |
| **Thomas (2021)** | single-center, parallel-arm, split-mouth, RCT## | Random order generated in RANDOM.org software. | The sequence was kept in sequentially numbered opaque sealed envelopes and concealed from the principal investigator. | Blinding was used at the data collection and analysis stages | 3 participants lost in MOPs group and 3 participants lost in control group due to irregular vist. | PPS |
| **Yadav (2025)** | single-centre, split-mouth,  RCT | Computer-generated random number table. | Allocation concealment was ensured using the sequentially numbered, opaque, sealed envelope (SNOSE) technique. | Blinding was used at the data collection and analysis stages | No loss | FAS |
| **Gulduren (2020)** | split-mouth,  RCT | NR | The sealed envelopes were shuffled thoroughly. | Participants and the operator was not blinded. The outcome examiner was blinded. | 1 participate lost in three groups respectively. | PPS |
| **Kumar (2024)** | RCT## | Randomization sequence was generated using Excel 2011 (Microsoft, Redmond, WA,USA). | The allocation was concealed by using sequentially numbered, opaque, sealed, and stapled envelopes. | Blinding was done at randomization and at the result assessment level. | No loss | FAS |
| **Fattori (2020)** | two arm parallel, RCT## | Number sequences generated using the Random function in Excel. | Before the randomization procedure, participants’ names were converted into letters and numbers by a different investigator to ensure allocation concealment. | Participants and the operator was not blinded. The outcome examiner was blinded to the subject’s assigned intervention. | 2 participants lost in MOPs group due to did not receive allocated intervention and space closure not completed. 4 participants lost in control group due to did not receive allocated intervention and space closure not completed/pregnancy. | PPS |
| **Babanouri (2020)** | single-center, split-mouth, triple-blind,  RCT## | the block randomization method (block length = 4) using the online RANDOM.ORG software.  The recruited patients were divided into two parallel groups with a 1:1 allocation ratio then the side of MOPs intervention in each subject was randomly determined with coin tossing. | Each random number was placed in a sealed opaque envelope and subsequently, each participant randomly picked one envelope. | Triple blinding design was employed. To ensure both the participants and clinicians were blinded to the clinical trial, a similar number of insertions were created only in the gingival tissue on the control side. MOPs intervention was performed by the first author. The outcome examiner was blinded. | 2 participants lost due to irregular attendance and debonding of the power arm. | PPS |

RCT: Randomized clinical trial; RCT ## : RCT with an allocation ratio of 1:1; NR: no report; FAS: Full analysis set; PPS: Per-Protocol set;

**Supplemental Figure 1. Meta-analysis of the distance of tooth movement**


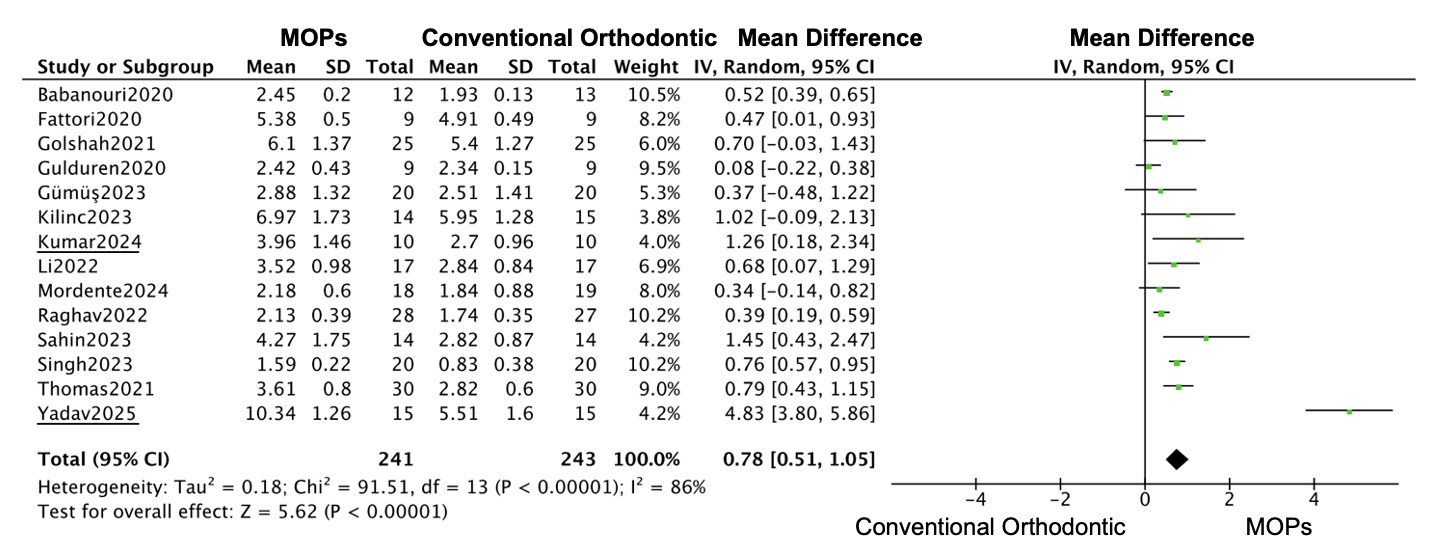


Supplemental Figure 1. Forest plot of the meta-analysis evaluating the effect of micro-osteoperforation (MOPs) on total orthodontic tooth movement distance. This plot compares the MOPs intervention group versus the conventional orthodontic treatment control group. The pooled mean difference (MD) with 95% confidence intervals (CI) was calculated using a random-effects model. In kummar’s and Yadav’s studies, datas are presented separately for the maxillary and mandible; in this case, the mean of these values is calculated with SPSS27.0 software.

**Supplemental Figure 2. Meta-analysis of the rate of tooth movement**


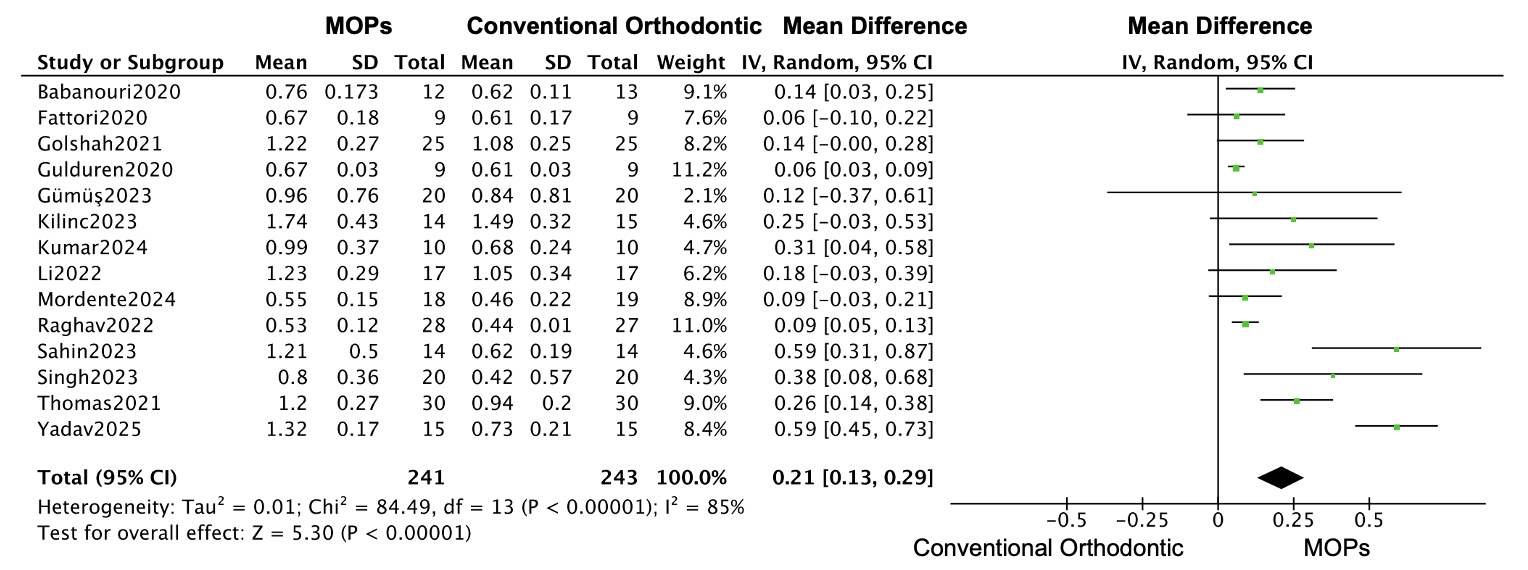


Supplemental Figure 2. Forest plot of the meta-analysis evaluating the effect of micro-osteoperforation (MOPs) on monthly orthodontic tooth movement rate. This plot compares the MOPs intervention group versus the conventional orthodontic treatment control group. The pooled mean difference (MD) with 95% confidence intervals (CI) was calculated using a random-effects model. In kummar’s and Yadav’s studies, datas are presented separately for the maxilla and mandible; in this case, the mean of these values is calculated with SPSS27.0 software.

**Supplemental Figure 3. Meta-analysis of the root resorption**

**
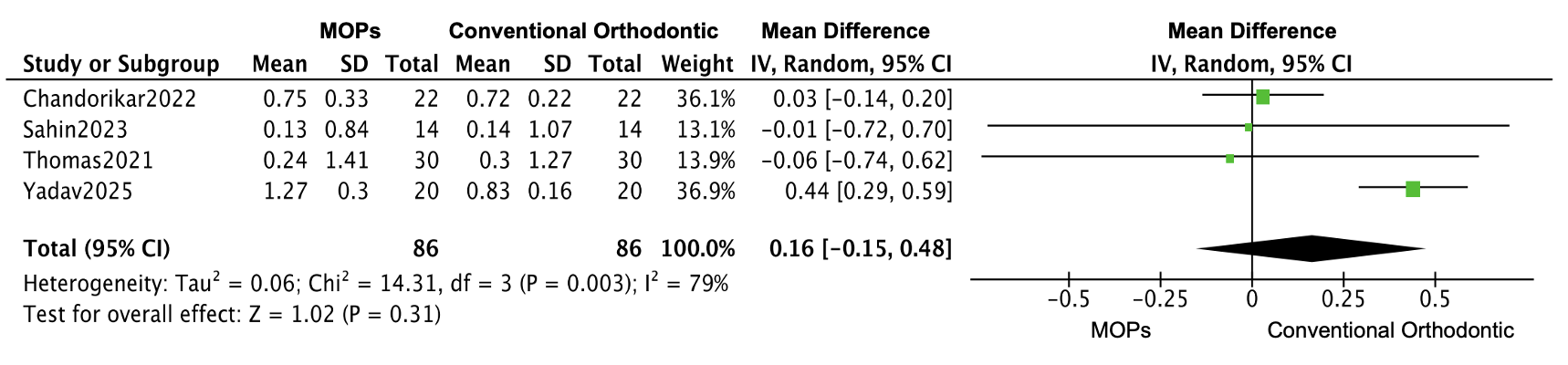
**

Supplemental Figure 3. Forest plot of the meta-analysis evaluating the effect of micro-osteoperforation (MOPs) on root resorption. This plot compares the intervention group receiving MOPs adjunctive to orthodontic treatment versus the control group receiving conventional orthodontic treatment alone. Root resorption is a continuous outcome measured as changes in root length. The pooled mean difference (MD) with 95% confidence intervals (CI) was calculated using a random-effects model. In Yadav’s studies, datas are presented separately for the maxilla and mandible; in this case, the mean of these values is calculated with SPSS27.0 software.

**Supplemental Figure 4. Subgroup analyses of main outcomes of interest (based on Maxillary & Mandibular respectively)**

| 1. **Meta-analysis of the distance of tooth movement (Maxillary)**   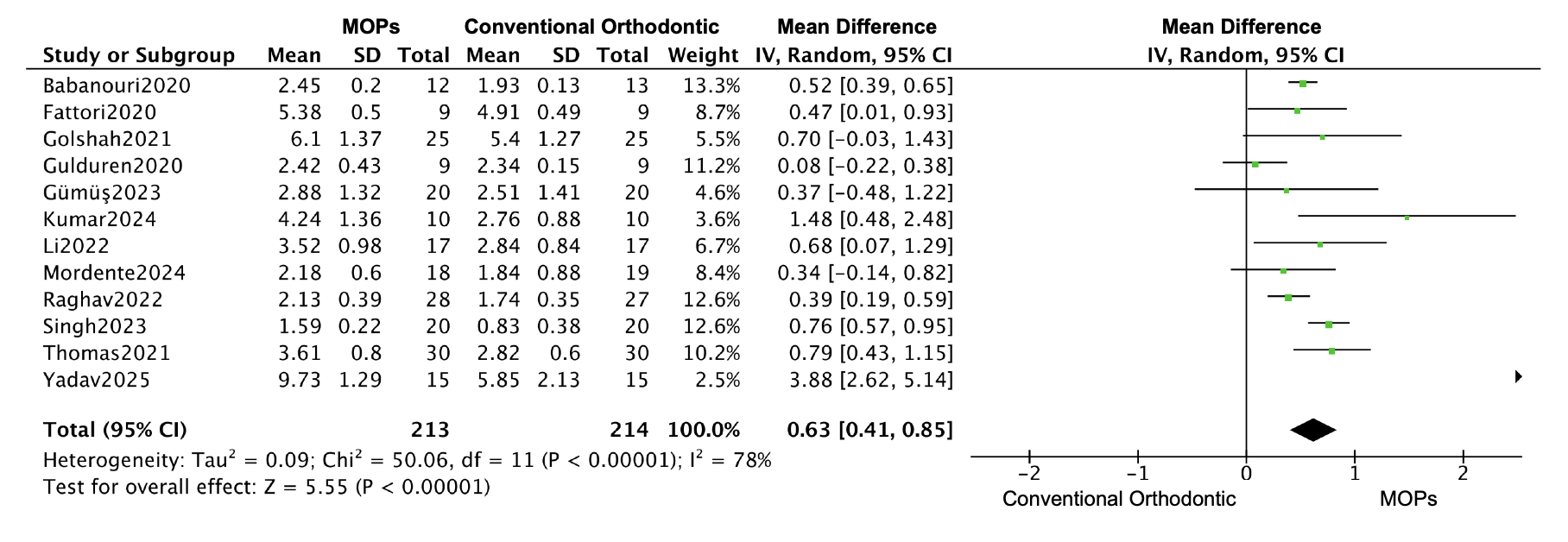 |
| --- |
| **(B) Meta-analysis of the distance of tooth movement (Mandibular)**  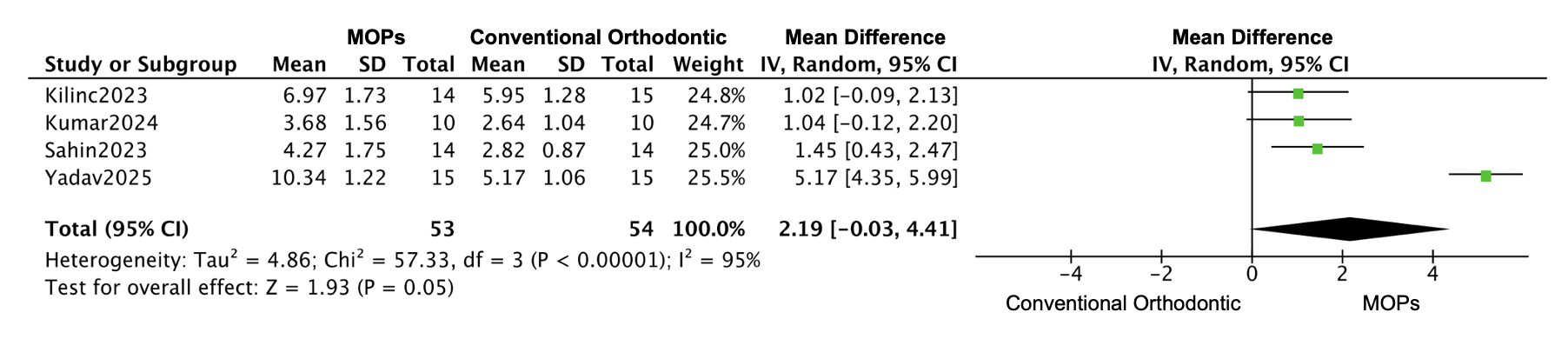 |
| **(C) Meta-analysis of the rate of tooth movement (Maxillary)**  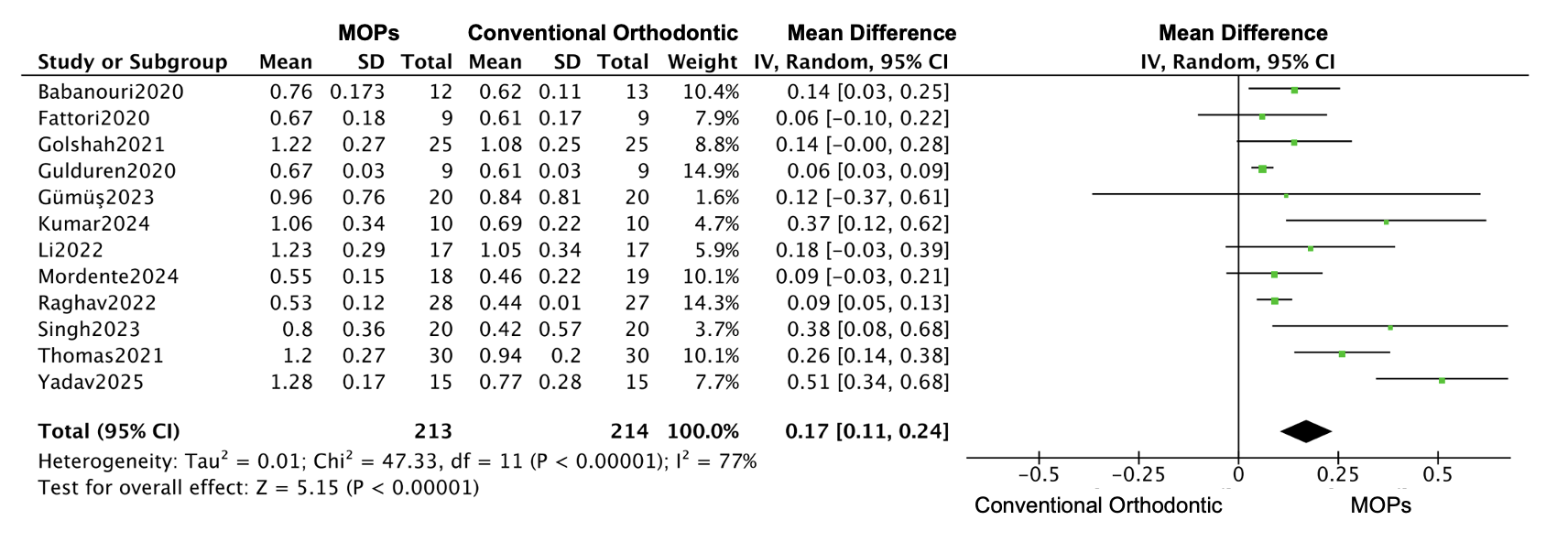 |
| **(D) Meta-analysis of the rate of tooth movement (Mandibular)**.  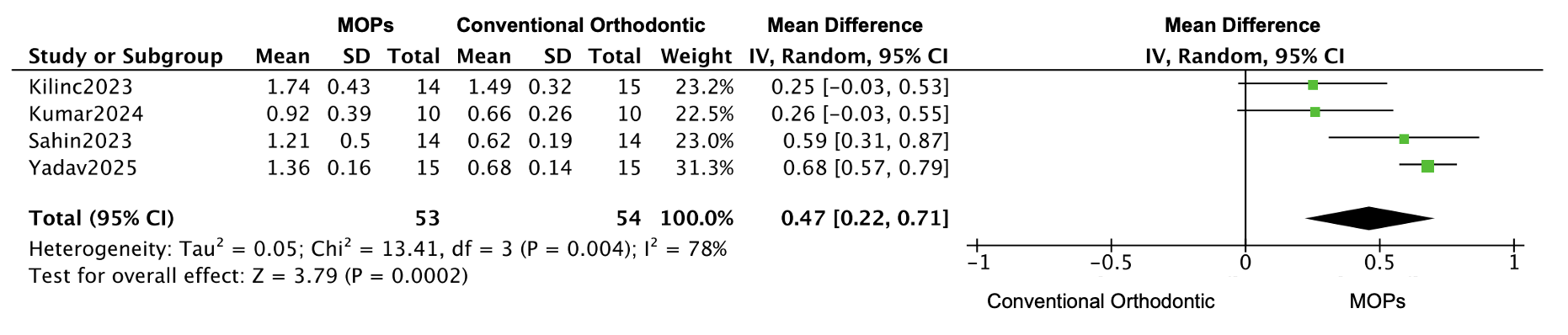 |
| **(E) Meta-analysis of the root resorption (Maxillary)**  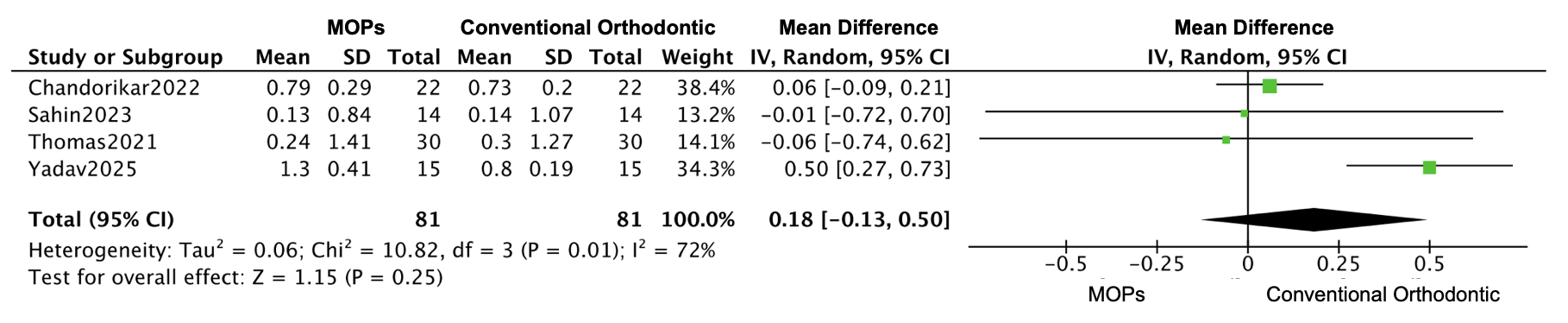 |
| **(F) Meta-analysis of the root resorption (Mandibular)**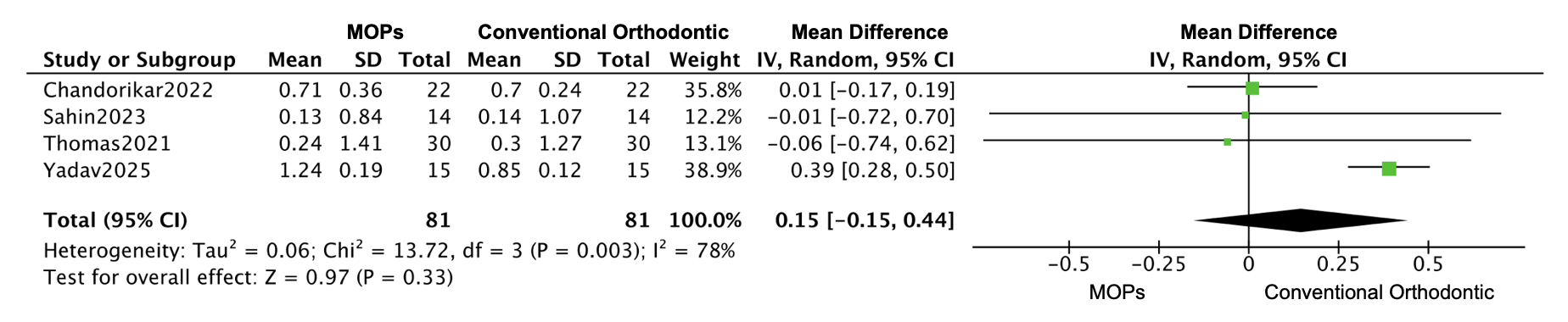 |

Supplemental Figure 4. Subgroup analyses of main outcomes of interest (based on Maxillary & Mandibular respectively)

(A) Forest plot of total tooth movement distance in maxillary. Comparison: MOPs vs. Conventional orthodontic treatment.

(B) Forest plot of total tooth movement distance in mandibular. Comparison: MOPs vs. Conventional orthodontic treatment.

(C) Forest plot of monthly tooth movement rate in maxillary. Comparison: MOPs vs. Conventional orthodontic treatment.

(D) Forest plot of monthly tooth movement rate in mandibular. Comparison: MOPs vs. Conventional orthodontic treatment.

(E) Forest plot of root resorption in maxillary. Comparison: MOPs vs. Conventional orthodontic treatment.

(F) Forest plot of root resorption in mandibular. Comparison: MOPs vs. Conventional orthodontic treatment.

**Supplemental Figure 5. Subgroup analyses of outcomes of interest (based on one/multiple MOPs and Maxillary & Mandibular)**

| **(A) Meta-analysis of the distance of tooth movement (one-time-MOPs and Maxillary)**  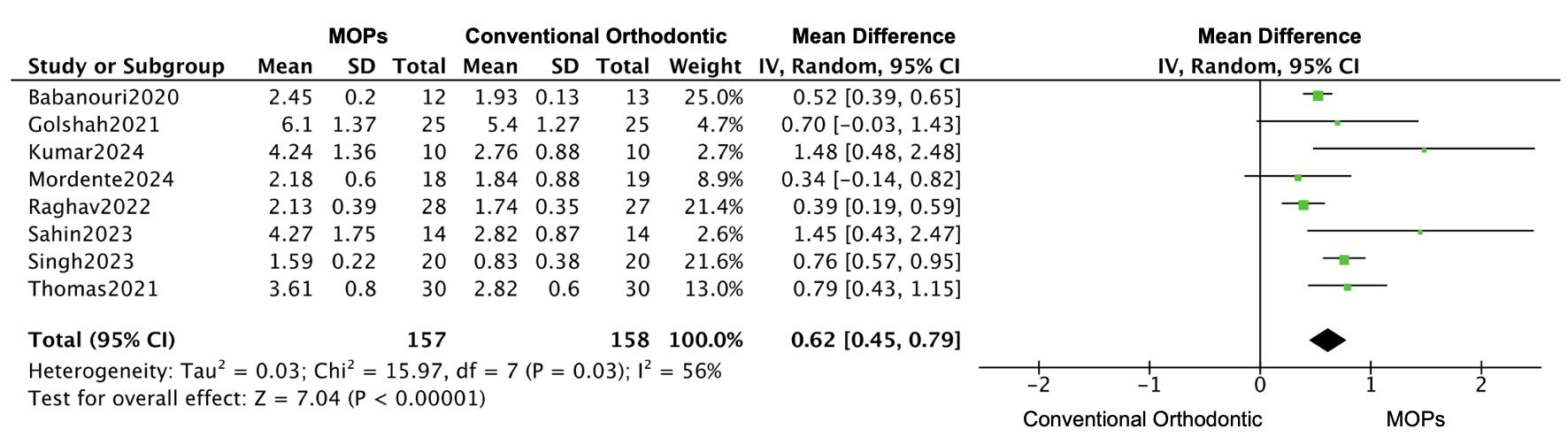 |
| --- |
| **(B) Meta-analysis of the distance of tooth movement (one-time-MOPs and Mandibular).**  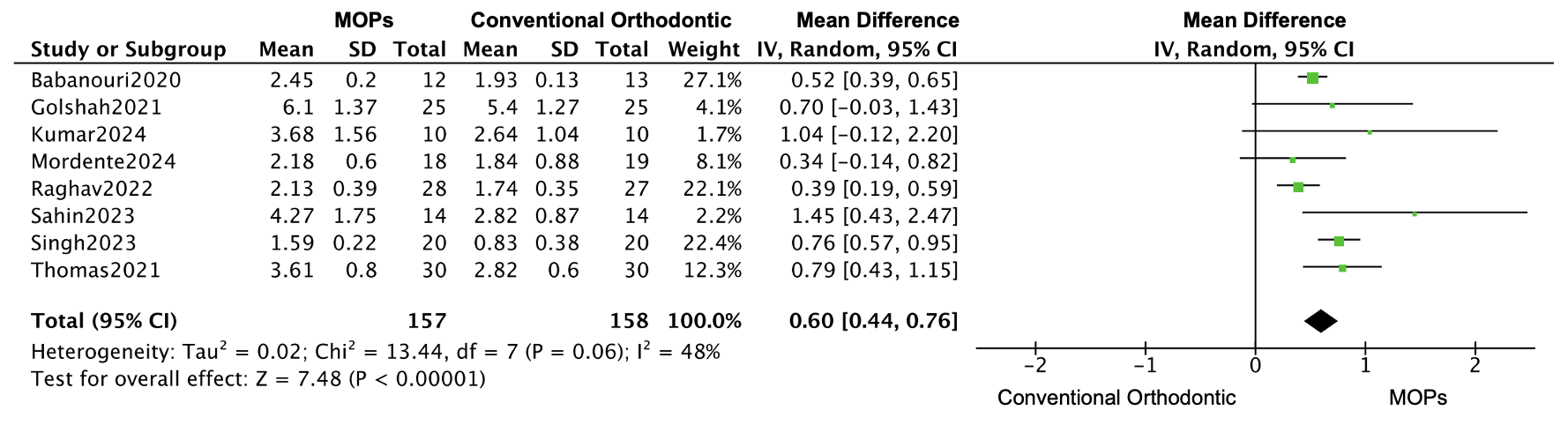 |
| **(C) Meta-analysis of the distance of tooth movement (multiple MOPs and Maxillary)**  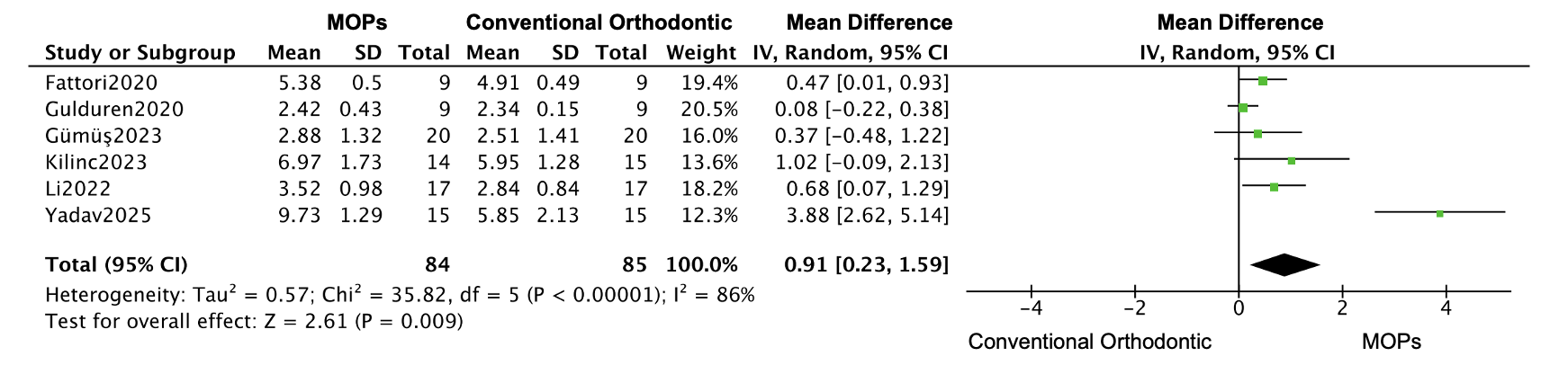 |
| **(D) Meta-analysis of the distance of tooth movement (multiple MOPs and Mandibular)**  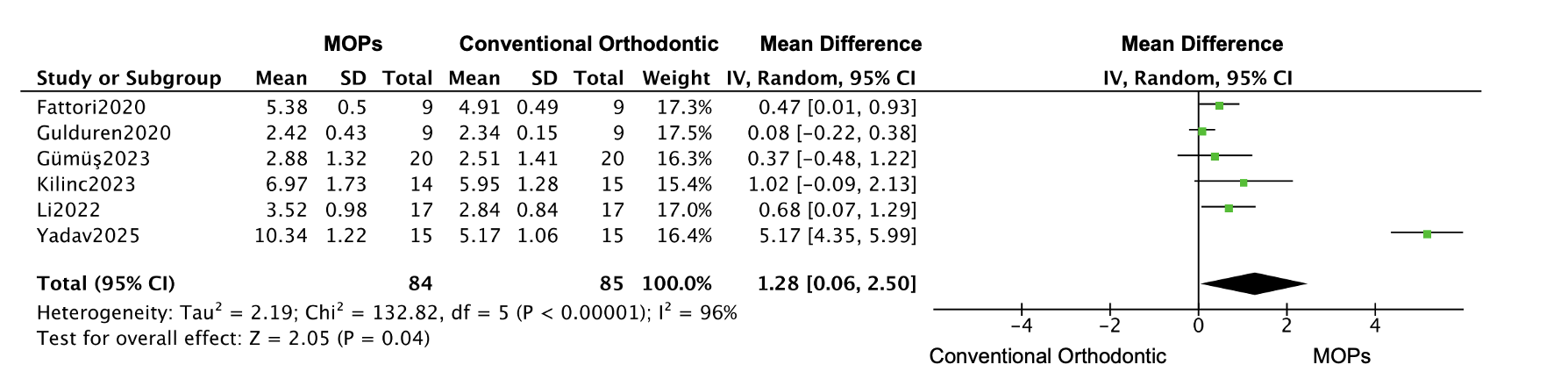 |
| **(E) Meta-analysis of the rate of tooth movement (one-time-MOPs and Maxillary)**  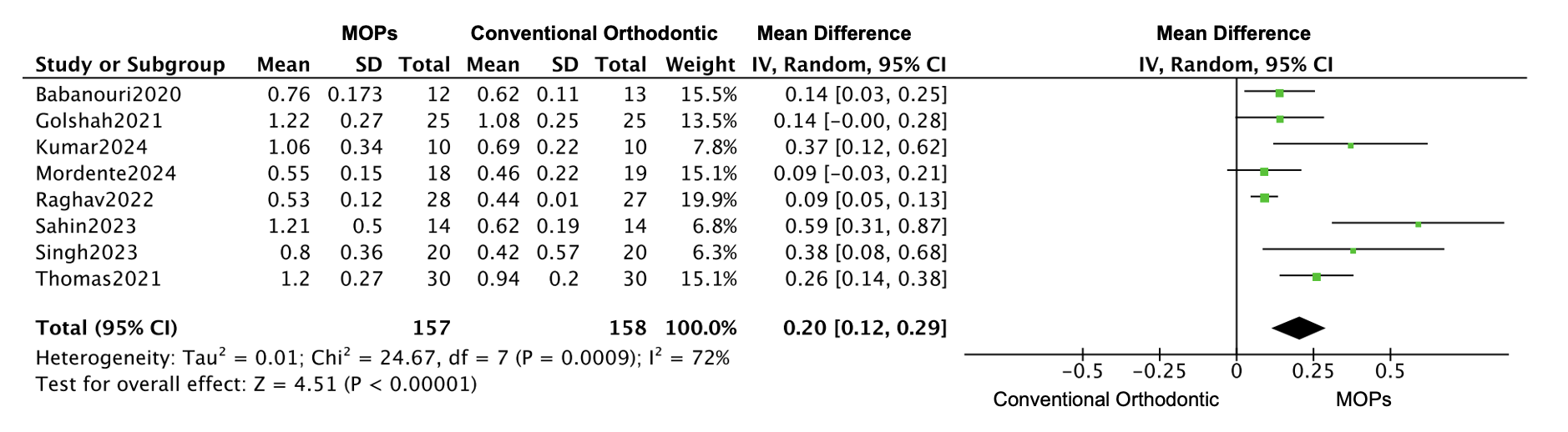 |
| **(F) Meta-analysis of the rate of tooth movement (one-time-MOPs and Mandibular)**  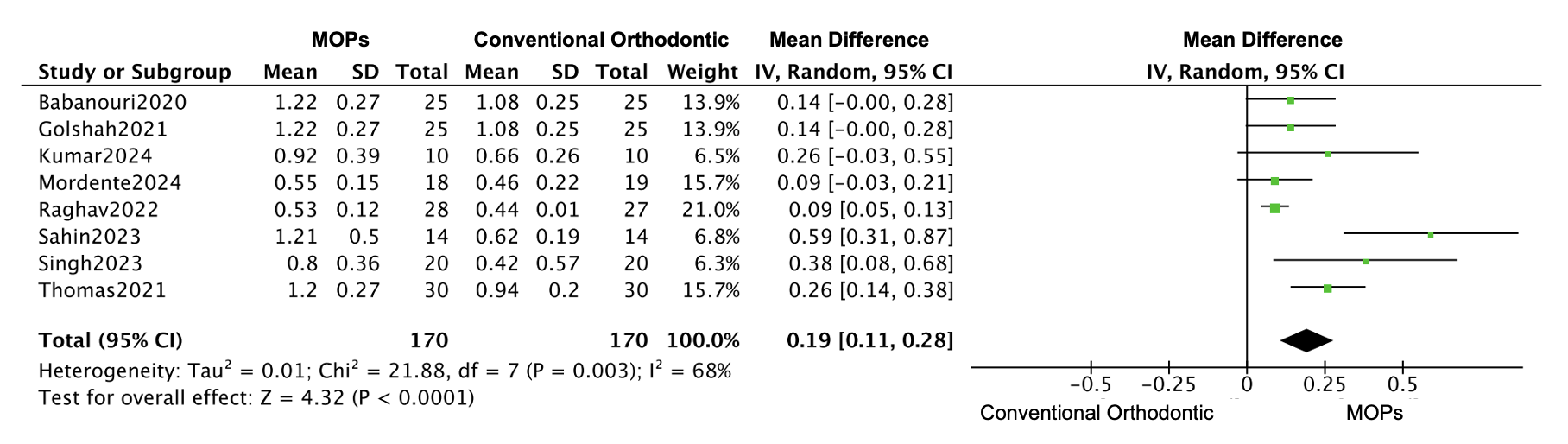 |
| **(G) Meta-analysis of the rate of tooth movement (multiple MOPs and Maxillary)**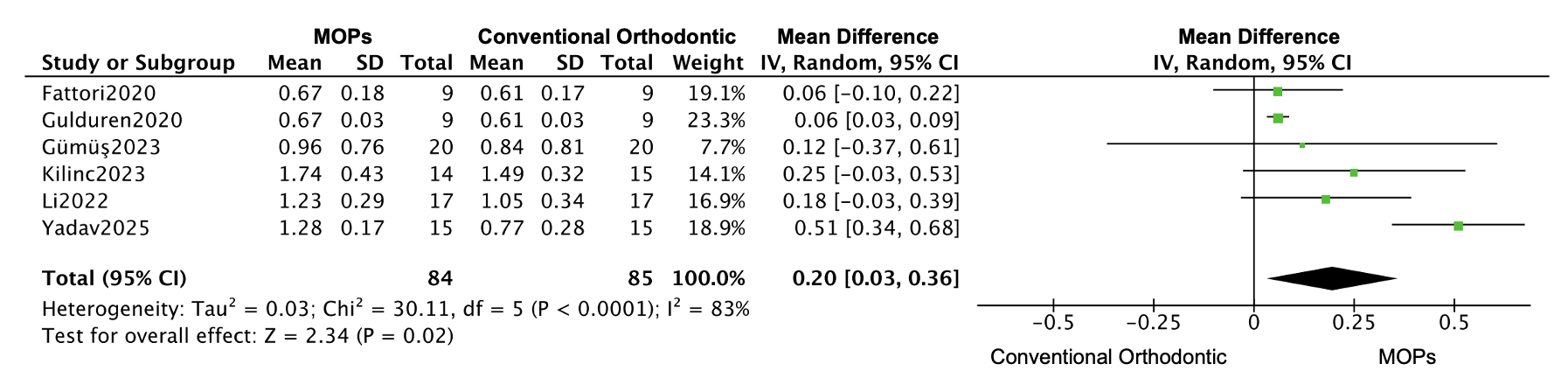 |
| **(H) Meta-analysis of the rate of tooth movement (multiple MOPs and Mandibular)**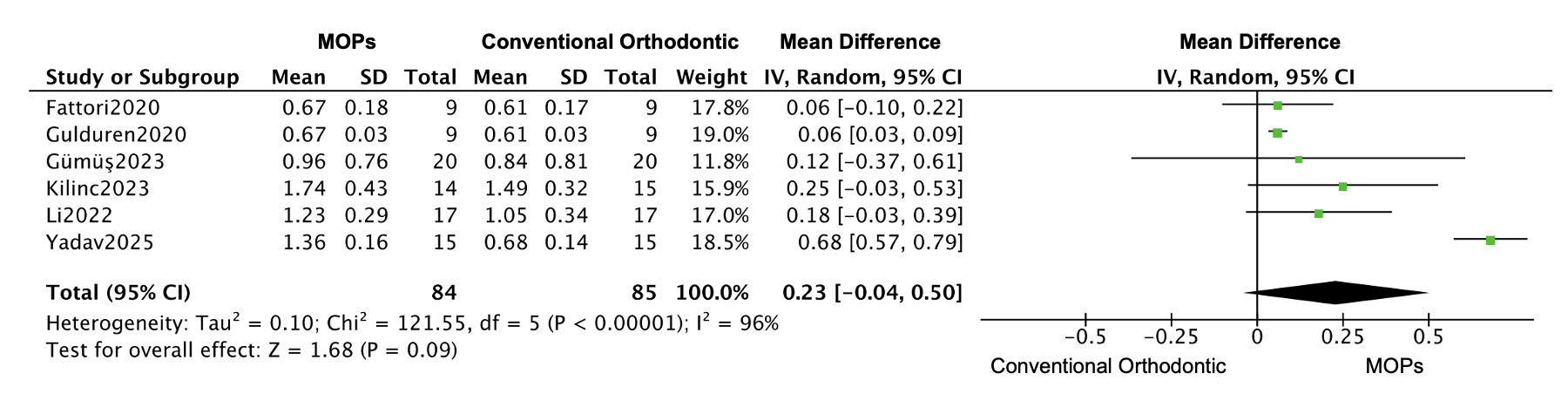 |

Supplemental Figure 5. Subgroup analyses of outcomes of interest (based on one/multiple MOPs and Maxillary & Mandibular)

(A) Total tooth movement distance with one-time MOPs in maxillary. Comparison: MOPs vs. Conventional orthodontic treatment.

(B) Total tooth movement distance with one-time MOPs in mandibular. Comparison: MOPs vs. Conventional orthodontic treatment.

(C) Total tooth movement distance with multiple MOPs in maxillary. Comparison: MOPs vs. Conventional orthodontic treatment.

(D) Total tooth movement distance with multiple MOPs in mandibular. Comparison: MOPs vs. Conventional orthodontic treatment.

(E) Monthly tooth movement rate with one-time MOPs in maxillary. Comparison: MOPs vs. Conventional orthodontic treatment.

(F) Monthly tooth movement rate with one-time MOPs in mandibular. Comparison: MOPs vs. Conventional orthodontic treatment.

(G) Monthly tooth movement rate with multiple MOPs in maxillary. Comparison: MOPs vs. Conventional orthodontic treatment.

(H) Monthly tooth movement rate with multiple MOPs in mandibular. Comparison: MOPs vs. Conventional orthodontic treatment.

**Supplemental Figure 6. Subgroup analyses of outcomes of interest (based on different instruments)**

| 1. **Meta-analysis of the distance of tooth movement (propel)**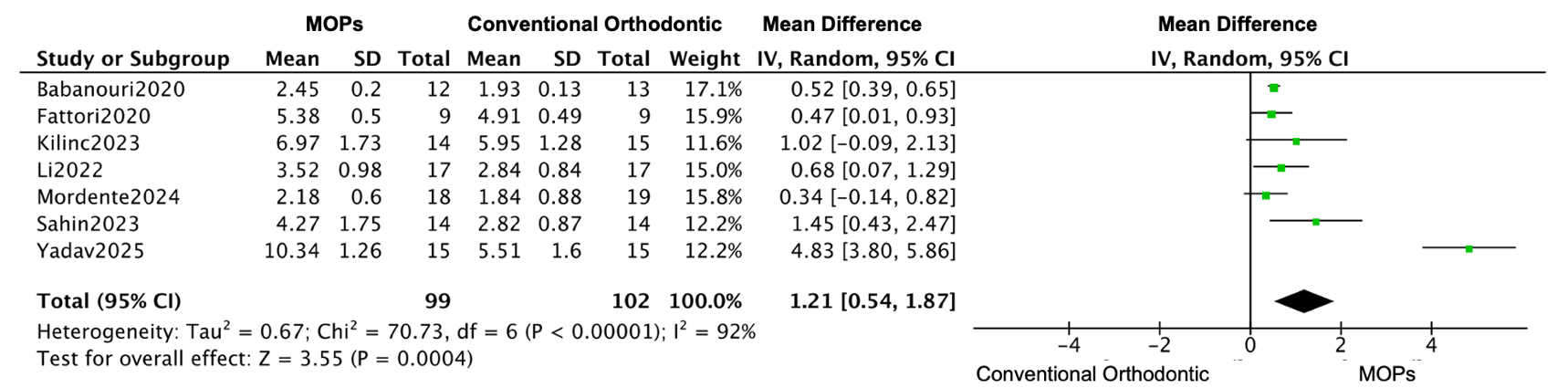 |
| --- |
| 1. **Meta-analysis of the distance of tooth movement (others)**   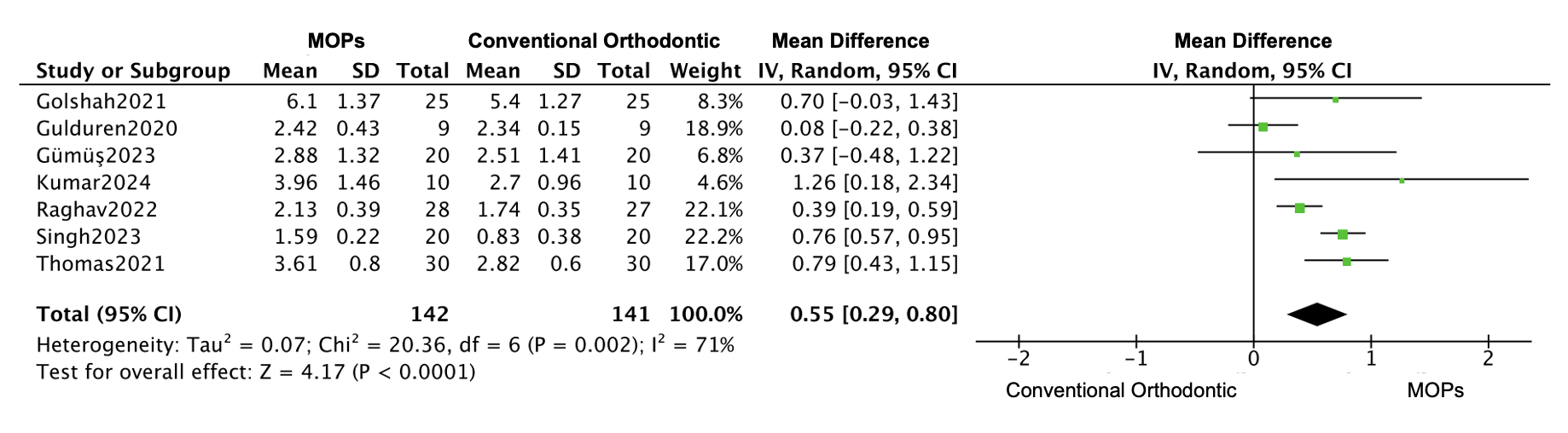 |

| **(C) Meta-analysis of the rate of tooth movement (propel)**  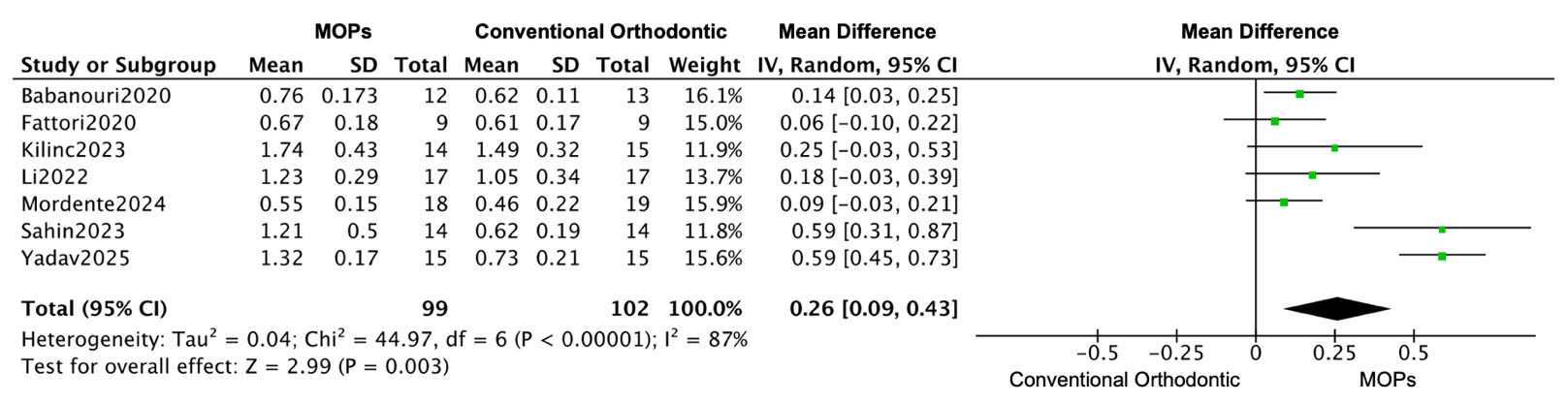 |
| --- |
| **(D) Meta-analysis of the rate of tooth movement (others)**  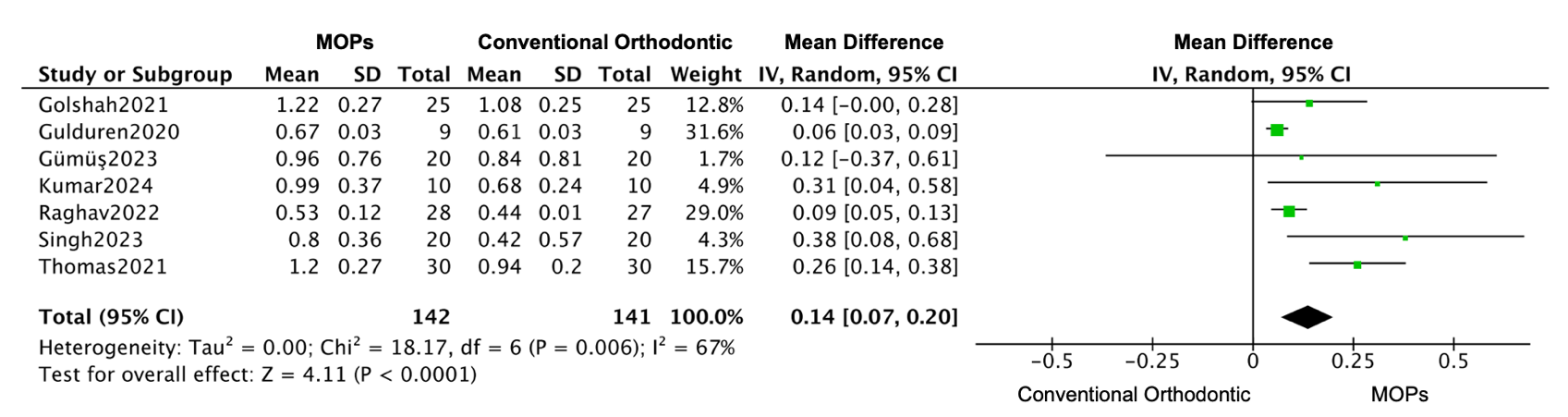 |

Supplemental Figure 6. Subgroup analyses of outcomes of interest (based on different instruments)

1. Total tooth movement distance using the Propel device. Comparison: MOPs vs. Conventional orthodontic treatment.
2. Total tooth movement distance using other instruments. Comparison: MOPs vs. Conventional orthodontic treatment.
3. Monthly tooth movement rate using the Propel device. Comparison: MOPs vs. Conventional orthodontic treatment.
4. Monthly tooth movement rate using other instruments. Comparison: MOPs vs. Conventional orthodontic treatment.

**Supplemental Figure 7. Funnel plots and Egger’s test of outcomes of interest**

| 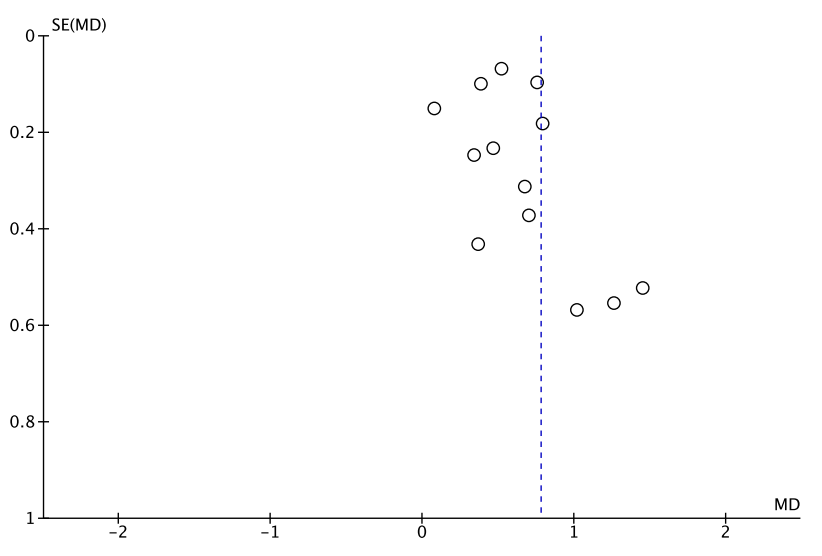 | 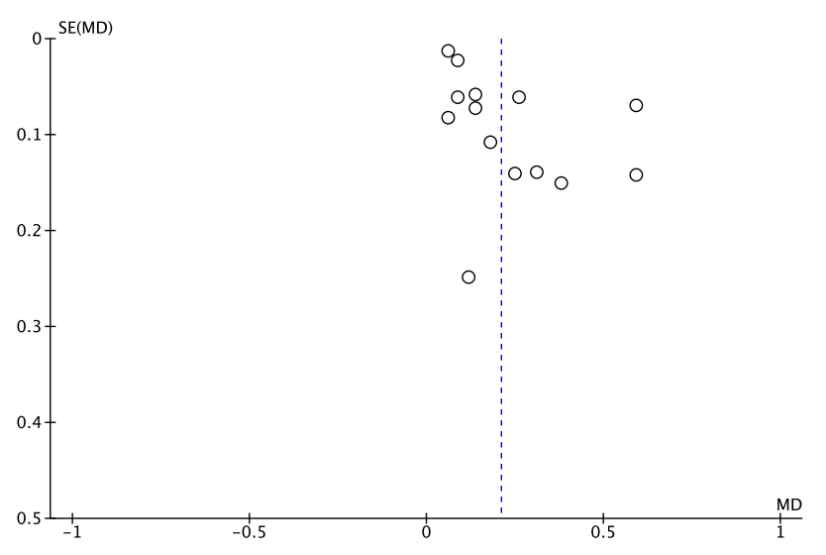 |
| --- | --- |
| Supplemental Figure 7A. Funnel plot for publication bias assessment of the primary outcome: the distance of tooth movement  The funnel plot was constructed based on the mean difference (MD) of total tooth movement distance between the MOPs group and the traditional orthodontics (non-MOPs) group (X-axis) and the standard error of MD (Y-axis). Each circle represents an individual included RCT. Asymmetry of the funnel plot was quantitatively evaluated by Egger’s linear regression test, with P < 0.05 indicating statistically significant publication bias. Egger’s test for this outcome: P = 0.000. | Supplemental Figure 7B. Funnel plot for publication bias assessment of the primary outcome: monthly rate of tooth movement  The funnel plot was constructed based on the mean difference (MD) of the monthly tooth movement rate between the MOPs group and the traditional orthodontics (non-MOPs) group (X-axis) and the standard error of MD (Y-axis). Each circle represents an individual included RCT. Asymmetry of the funnel plot was quantitatively evaluated by Egger’s linear regression test, with P < 0.05 indicating statistically significant publication bias. Egger’s test for this outcome: P = 0.001. |
| 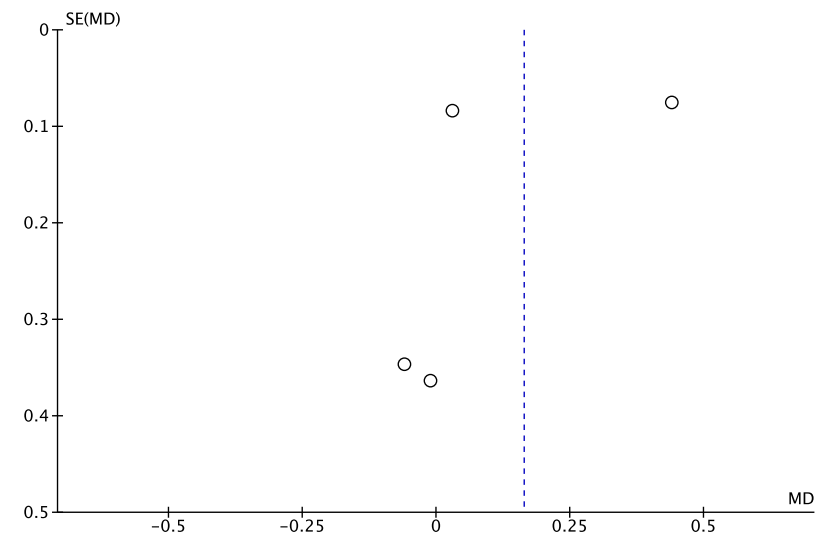 | |
| Supplemental Figure 7C. Funnel plot for publication bias assessment of the secondary outcome: root resorption  The funnel plot was constructed based on the mean difference (MD) of root resorption between the MOPs group and the traditional orthodontics (non-MOPs) group (X-axis) and the standard error of MD (Y-axis). Each circle represents an individual included RCT. Asymmetry of the funnel plot was quantitatively evaluated by Egger’s linear regression test, with P < 0.05 indicating statistically significant publication bias. Egger’s test for this outcome: P = 0.043. | |
| 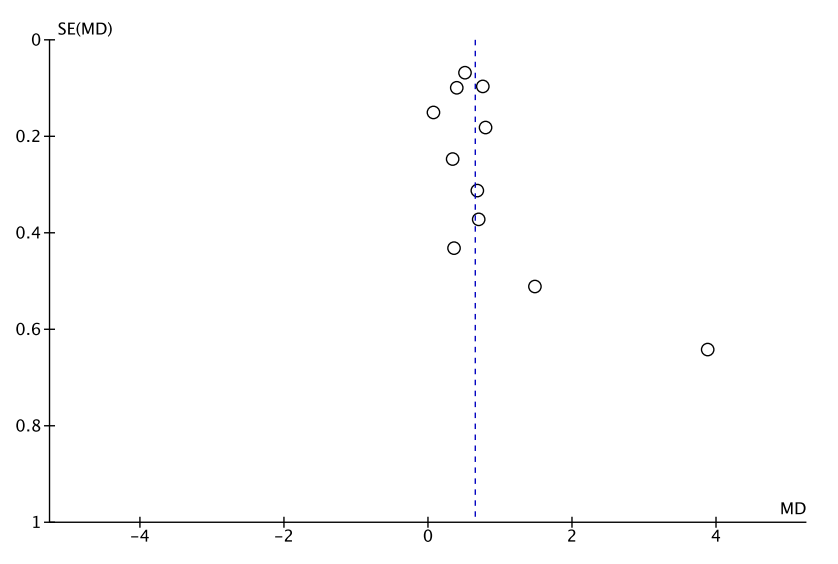 | 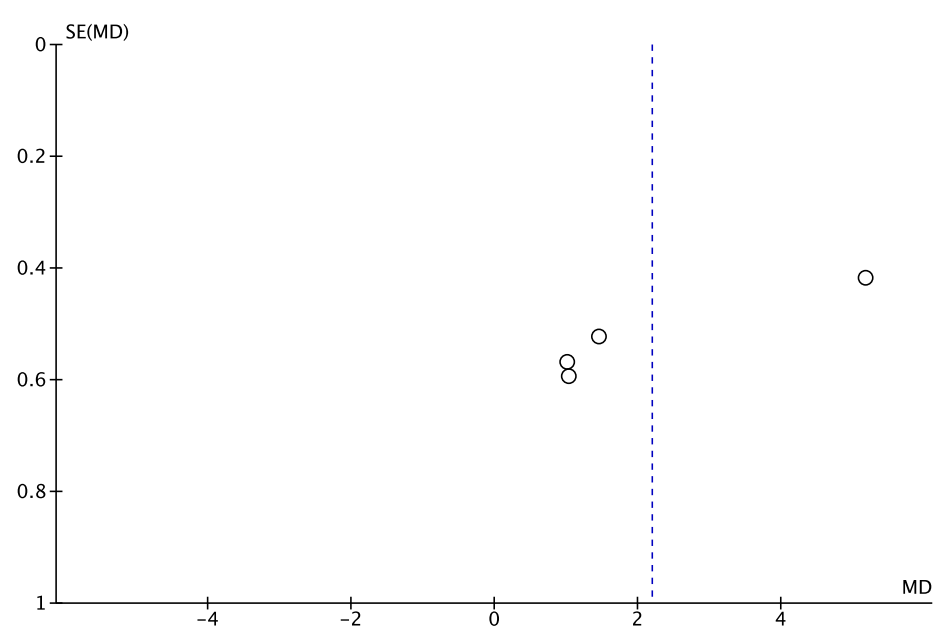 |
| Supplemental Figure 7D. Funnel plot for publication bias assessment of the subgroup outcome: the distance of tooth movement in maxillary  The funnel plot was constructed based on the mean difference (MD) of maxillary tooth movement distance between the MOPs group and the traditional orthodontics (non-MOPs) group (X-axis) and the standard error of MD (Y-axis). Each circle represents an individual included RCT. Asymmetry of the funnel plot was quantitatively evaluated by Egger’s linear regression test, with P < 0.05 indicating statistically significant publication bias. Egger’s test for this outcome: P = 0.000. | Supplemental Figure 7E. Funnel plot for publication bias assessment of the subgroup outcome: the distance of tooth movement in mandibular  The funnel plot was constructed based on the mean difference (MD) of mandibular tooth movement distance between the MOPs group and the traditional orthodontics (non-MOPs) group (X-axis) and the standard error of MD (Y-axis). Each circle represents an individual included RCT. Asymmetry of the funnel plot was quantitatively evaluated by Egger’s linear regression test, with P < 0.05 indicating statistically significant publication bias. Egger’s test for this outcome:  P = 0.129. |
| 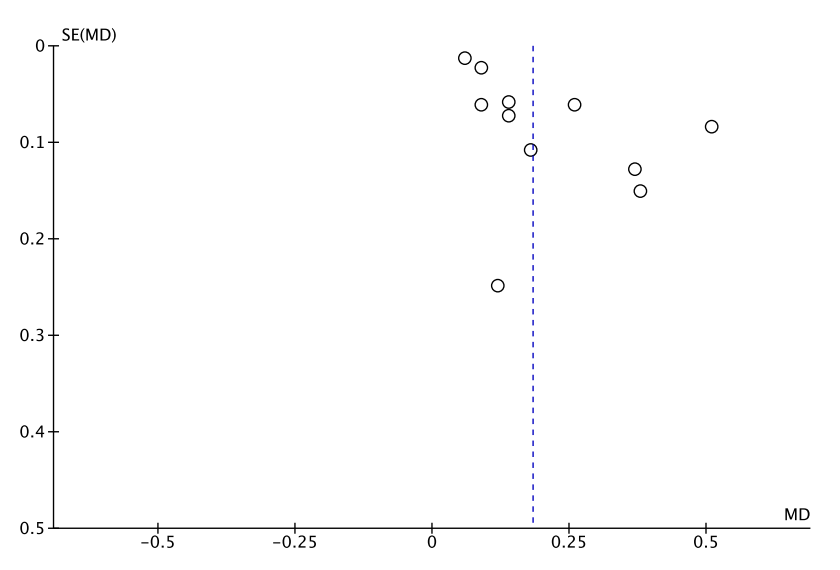 | 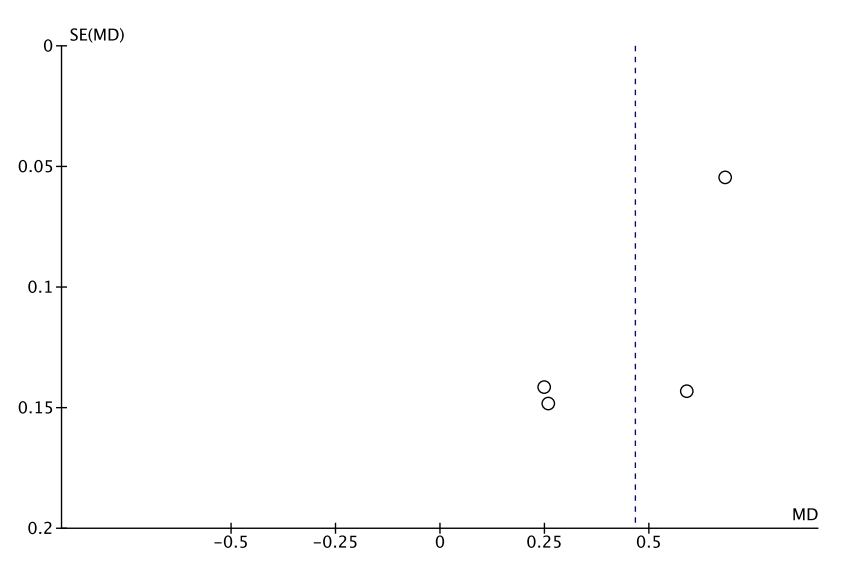 |
| Supplemental Figure 7F. Funnel plot for publication bias assessment of the subgroup outcome: monthly tooth movement rate in maxillary  The funnel plot was constructed based on the mean difference (MD) of maxillary tooth movement rate between the MOPs group and the traditional orthodontics (non-MOPs) group (X-axis) and the standard error of MD (Y-axis). Each circle represents an individual included RCT. Asymmetry of the funnel plot was quantitatively evaluated by Egger’s linear regression test, with P < 0.05 indicating statistically significant publication bias. Egger’s test for this outcome: P = 0.002. | Supplemental Figure 7G. Funnel plot for publication bias assessment of the subgroup outcome: monthly tooth movement rate in the mandibular  The funnel plot was constructed based on the mean difference (MD) of mandibular tooth movement rate between the MOPs group and the traditional orthodontics (non-MOPs) group (X-axis) and the standard error of MD (Y-axis). Each circle represents an individual included RCT. Asymmetry of the funnel plot was quantitatively evaluated by Egger’s linear regression test, with P < 0.05 indicating statistically significant publication bias. Egger’s test for this outcome: P = 0.373. |
| 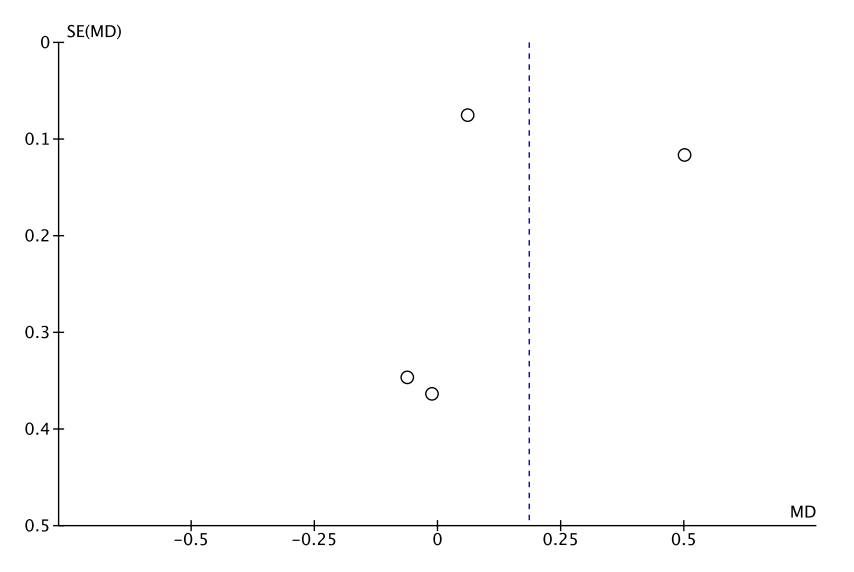 | 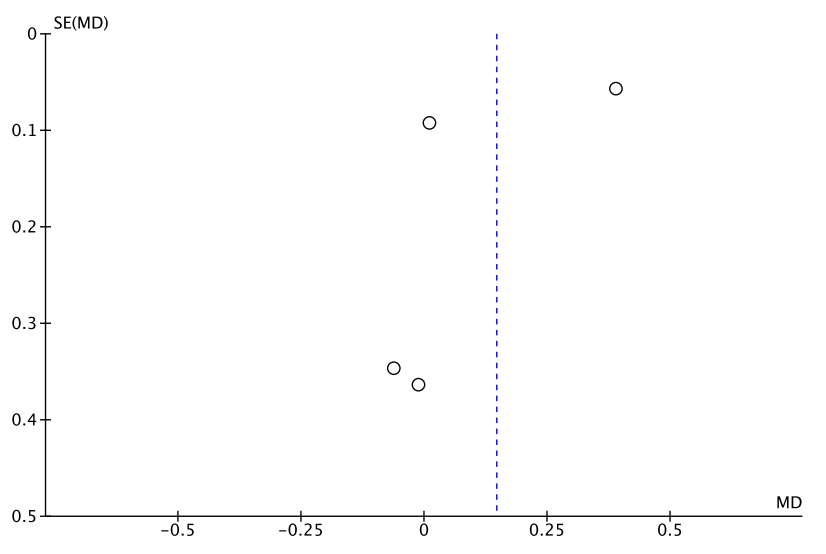 |
| Supplemental Figure 7H. Funnel plot for publication bias assessment of the subgroup outcome: root resorption in maxillary  The funnel plot was constructed based on the mean difference (MD) of maxillary root resorption between the MOPs group and the traditional orthodontics (non-MOPs) group (X-axis) and the standard error of MD (Y-axis). Each circle represents an individual included RCT. Asymmetry of the funnel plot was quantitatively evaluated by Egger’s linear regression test, with P < 0.05 indicating statistically significant publication bias. Egger’s test for this outcome: P = 0.049. | Supplemental Figure 7I. Funnel plot for publication bias assessment of the subgroup outcome: root resorption in mandibular  The funnel plot was constructed based on the mean difference (MD) of mandibular root resorption between the MOPs group and the traditional orthodontics (non-MOPs) group (X-axis) and the standard error of MD (Y-axis). Each circle represents an individual included RCT. Asymmetry of the funnel plot was quantitatively evaluated by Egger’s linear regression test, with P < 0.05 indicating statistically significant publication bias. Egger’s test for this outcome: P = 0.039. |
| 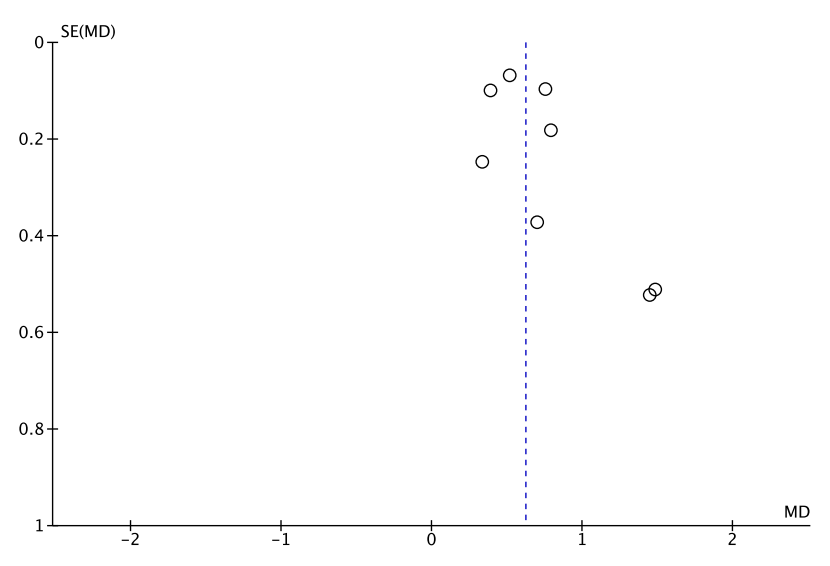 | 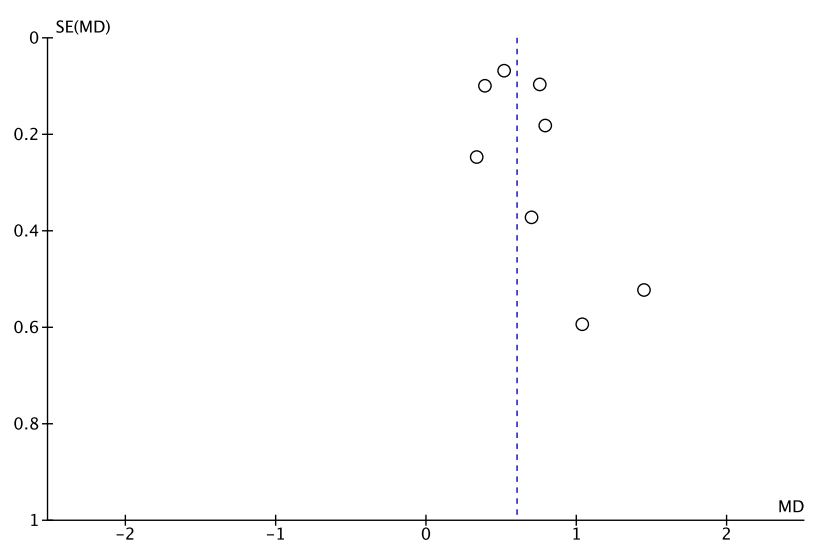 |
| Supplemental Figure 7J. Funnel plot for publication bias assessment of the subgroup outcome: the distance of tooth movement with one-time MOPs in maxillary  The funnel plot was constructed based on the mean difference (MD) of maxillary tooth movement distance between the one-time MOPs group and the traditional orthodontics (non-MOPs) group (X-axis) and the standard error of MD (Y-axis). Each circle represents an individual included RCT. Asymmetry of the funnel plot was quantitatively evaluated by Egger’s linear regression test, with P < 0.05 indicating statistically significant publication bias. Egger’s test for this outcome: P = 0.000. | Supplemental Figure 7K. Funnel plot for publication bias assessment of the subgroup outcome: the distance of tooth movement with one-time MOPs in mandibular  The funnel plot was constructed based on the mean difference (MD) of mandibular tooth movement distance between the one-time MOPs group and the traditional orthodontics (non-MOPs) group (X-axis) and the standard error of MD (Y-axis). Each circle represents an individual included RCT. Asymmetry of the funnel plot was quantitatively evaluated by Egger’s linear regression test, with P < 0.05 indicating statistically significant publication bias. Egger’s test for this outcome: P = 0.000. |
| 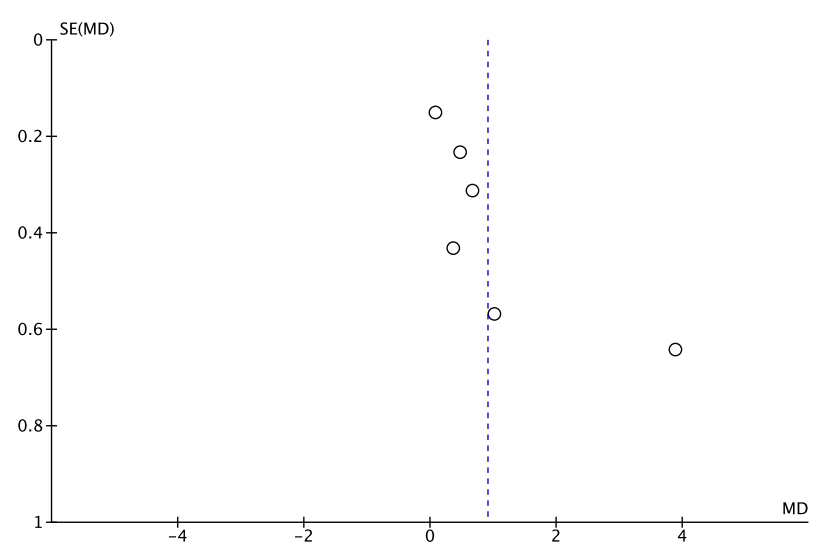 | 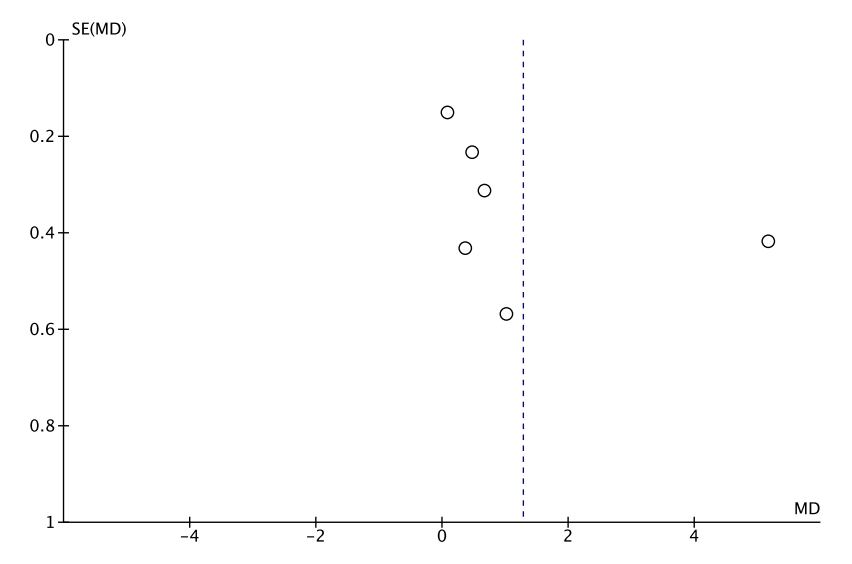 |
| Supplemental Figure 7L. Funnel plot for publication bias assessment of the subgroup outcome: the distance of tooth movement with multiple MOPs in maxillary  The funnel plot was constructed based on the mean difference (MD) of maxillary tooth movement distance between the multiple MOPs group and the traditional orthodontics (non-MOPs) group (X-axis) and the standard error of MD (Y-axis). Each circle represents an individual included RCT. Asymmetry of the funnel plot was quantitatively evaluated by Egger’s linear regression test, with P < 0.05 indicating statistically significant publication bias. Egger’s test for this outcome: P = 0.004. | Supplemental Figure 7M. Funnel plot for publication bias assessment of the subgroup outcome: the distance of tooth movement with multiple MOPs in mandibular  The funnel plot was constructed based on the mean difference (MD) of mandibular tooth movement distance between the multiple MOPs group and the traditional orthodontics (non-MOPs) group (X-axis) and the standard error of MD (Y-axis). Each circle represents an individual included RCT. Asymmetry of the funnel plot was quantitatively evaluated by Egger’s linear regression test, with P < 0.05 indicating statistically significant publication bias. Egger’s test for this outcome: P = 0.018. |
| 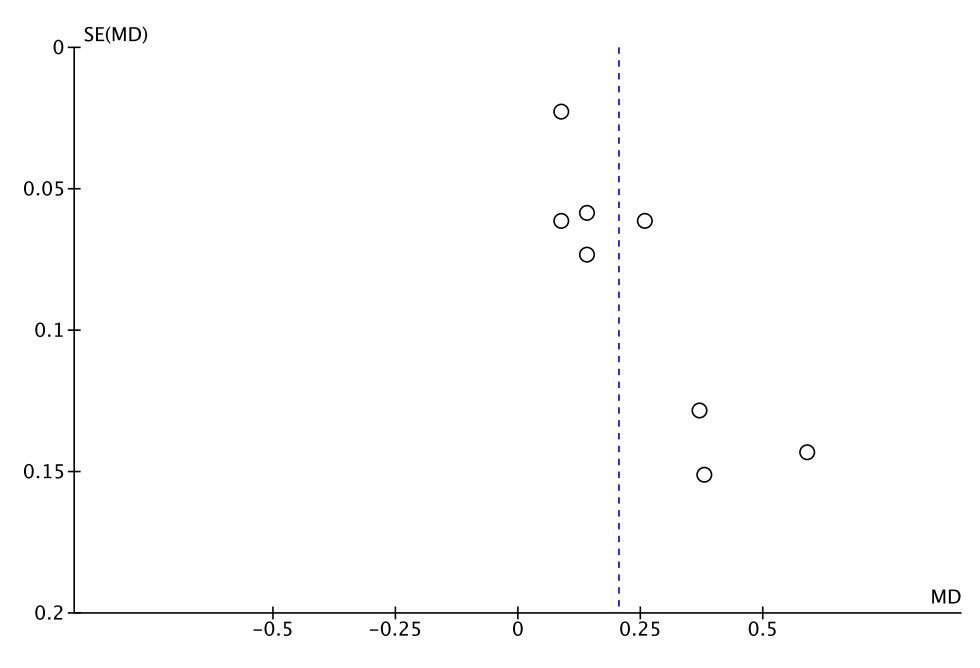 | 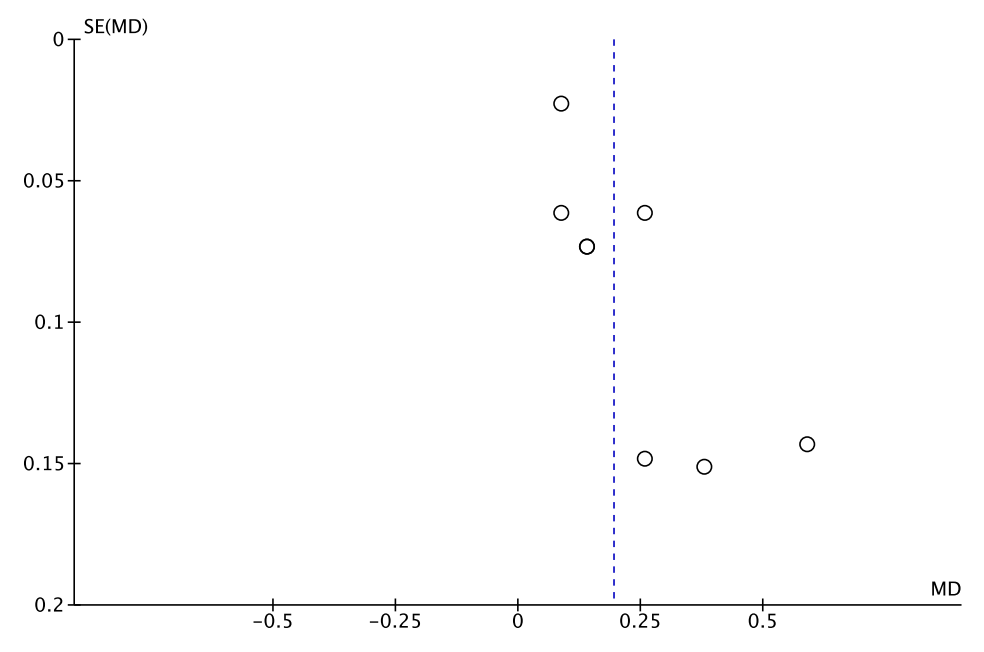 |
| Supplemental Figure 7N. Funnel plot for publication bias assessment of the subgroup outcome: monthly tooth movement rate with one-time MOPs in maxillary  The funnel plot was constructed based on the mean difference (MD) of maxillary tooth movement rate between the one-time MOPs group and the traditional orthodontics (non-MOPs) group (X-axis) and the standard error of MD (Y-axis). Each circle represents an individual included RCT. Asymmetry of the funnel plot was quantitatively evaluated by Egger’s linear regression test, with P < 0.05 indicating statistically significant publication bias. Egger’s test for this outcome: P = 0.034. | Supplemental Figure 7O. Funnel plot for publication bias assessment of the subgroup outcome: monthly tooth movement rate with multiple MOPs in maxillary  The funnel plot was constructed based on the mean difference (MD) of mandibular tooth movement rate between the multiple MOPs group and the traditional orthodontics (non-MOPs) group (X-axis) and the standard error of MD (Y-axis). Each circle represents an individual included RCT. Asymmetry of the funnel plot was quantitatively evaluated by Egger’s linear regression test, with P < 0.05 indicating statistically significant publication bias. Egger’s test for this outcome: P = 0.026. |
| 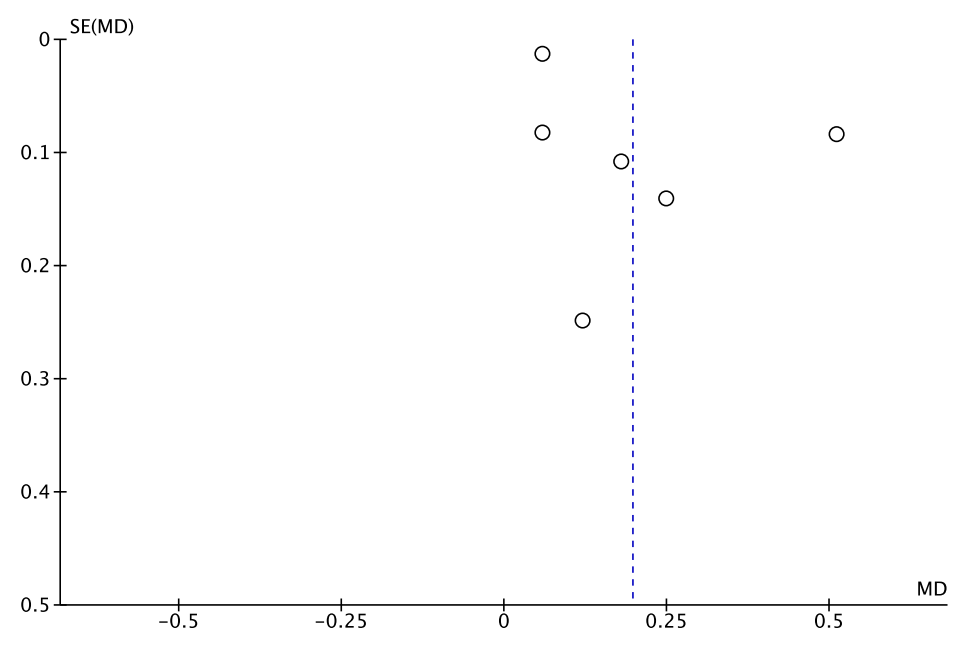 | 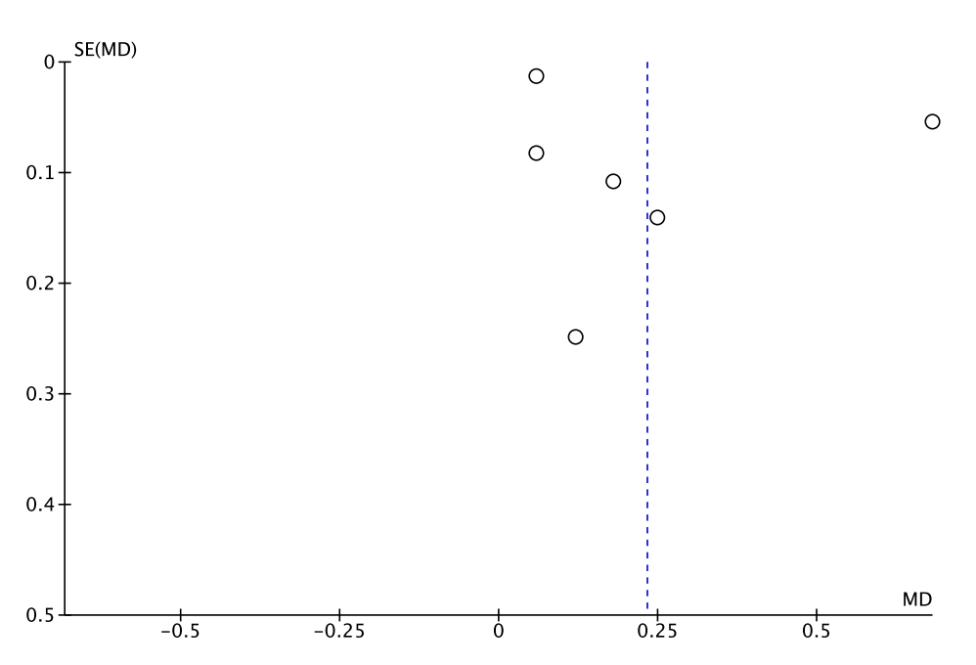 |
| Supplemental Figure 7P. Funnel plot for publication bias assessment of the subgroup outcome: monthly tooth movement rate with multiple MOPs in maxillary  The funnel plot was constructed based on the mean difference (MD) of maxillary tooth movement rate between the multiple MOPs group and the traditional orthodontics (non-MOPs) group (X-axis) and the standard error of MD (Y-axis). Each circle represents an individual included RCT. Asymmetry of the funnel plot was quantitatively evaluated by Egger’s linear regression test, with P < 0.05 indicating statistically significant publication bias. Egger’s test for this outcome: P = 0.025. | Supplemental Figure 7R. Funnel plot for publication bias assessment of the subgroup outcome: monthly tooth movement rate with multiple MOPs in mandibular  The funnel plot was constructed based on the mean difference (MD) of mandibular tooth movement rate between the multiple MOPs group and the traditional orthodontics (non-MOPs) group (X-axis) and the standard error of MD (Y-axis). Each circle represents an individual included RCT. Asymmetry of the funnel plot was quantitatively evaluated by Egger’s linear regression test, with P < 0.05 indicating statistically significant publication bias. Egger’s test for this outcome: P = 0.136. |
